# A degradome-based prognostic signature that correlates with immune infiltration and tumor mutation burden in breast cancer

**DOI:** 10.3389/fimmu.2023.1140993

**Published:** 2023-03-13

**Authors:** Yulou Luo, Yinghui Ye, Yan Chen, Chenguang Zhang, Yutian Sun, Chengwei Wang, Jianghua Ou

**Affiliations:** ^1^ Department of Breast Surgery, Affiliated Tumor Hospital of Xinjiang Medical University, Urumqi, China; ^2^ Department of Laboratory Medicine, Peking University Shenzhen Hospital, Shenzhen, China; ^3^ Department of Biochemistry and Molecular Biology, School of Basic Medical Sciences, Xinjiang Medical University, Urumqi, China; ^4^ Department of Medical Oncology, Sichuan Cancer Hospital and Institute, Sichuan Cancer Center, School of Medicine, University of Electronic Science and Technology of China, Chengdu, China; ^5^ Cancer Research Institute of Xinjiang Uygur Autonomous Region, Affiliated Tumor Hospital of Xinjiang Medical University, Urumqi, China

**Keywords:** degradome, prognostic signature, tumour mutation burden, immune infiltration, immunotherapy, breast cancer

## Abstract

**Introduction:**

Female breast cancer is the most common malignancy worldwide, with a high disease burden. The degradome is the most abundant class of cellular enzymes that play an essential role in regulating cellular activity. Dysregulation of the degradome may disrupt cellular homeostasis and trigger carcinogenesis. Thus we attempted to understand the prognostic role of degradome in breast cancer by means of establishing a prognostic signature based on degradome-related genes (DRGs) and assessed its clinical utility in multiple dimensions.

**Methods:**

A total of 625 DRGs were obtained for analysis. Transcriptome data and clinical information of patients with breast cancer from TCGA-BRCA, METABRIC and GSE96058 were collected. NetworkAnalyst and cBioPortal were also utilized for analysis. LASSO regression analysis was employed to construct the degradome signature. Investigations of the degradome signature concerning clinical association, functional characterization, mutation landscape, immune infiltration, immune checkpoint expression and drug priority were orchestrated. Cell phenotype assays including colony formation, CCK8, transwell and wound healing were conducted in MCF-7 and MDA-MB-435S breast cancer cell lines, respectively.

**Results:**

A 10-gene signature was developed and verified as an independent prognostic predictor combined with other clinicopathological parameters in breast cancer. The prognostic nomogram based on risk score (calculated based on the degradome signature) showed favourable capability in survival prediction and advantage in clinical benefit. High risk scores were associated with a higher degree of clinicopathological events (T4 stage and HER2-positive) and mutation frequency. Regulation of toll-like receptors and several cell cycle promoting activities were upregulated in the high-risk group. PIK3CA and TP53 mutations were dominant in the low- and high-risk groups, respectively. A significantly positive correlation was observed between the risk score and tumor mutation burden. The infiltration levels of immune cells and the expressions of immune checkpoints were significantly influenced by the risk score. Additionally, the degradome signature adequately predicted the survival of patients undergoing endocrinotherapy or radiotherapy. Patients in the low-risk group may achieve complete response after the first round of chemotherapy with cyclophosphamide and docetaxel, whereas patients in the high-risk group may benefit from 5-flfluorouracil. Several regulators of the PI3K/AKT/mTOR signaling pathway and the CDK family/PARP family were identified as potential molecular targets in the low- and high-risk groups, respectively. In vitro experiments further revealed that the knockdown of ABHD12 and USP41 significantly inhibit the proliferation, invasion and migration of breast cancer cells.

**Conclusion:**

Multidimensional evaluation verified the clinical utility of the degradome signature in predicting prognosis, risk stratification and guiding treatment for patients with breast cancer.

## 1 Introduction

According to GLOBOCAN 2020 statistics, female breast cancer is the most common malignancy worldwide, with approximately 2.3 million new cases reported annually, and is the fifth leading cause of cancer-related death (6.9%), thus imposing a huge disease burden worldwide (1). Breast cancer originates from mammary gland epithelial cells. The most common types of breast cancer are infiltrating ductal carcinoma (IDC) and infiltrating lobular carcinoma (ILC). Other types include ductal carcinoma *in situ* (DCIS), lobular carcinoma *in situ* (LCIS), mucinous carcinoma and medullary carcinoma. Breast cancer is a major focus of anti-cancer research. Since the beginning of the 21^st^ century, remarkable progress has been achieved in the treatment of breast cancer. Some patients with breast cancer significantly benefit from targeted therapy and immune checkpoint blockade (2). Despite substantial progress, some challenges remain unresolved at present, such as chemotherapy resistance, unperceived distant metastasis, triple-negative breast cancer (TNBC) treatment and the unavailability of sufficient molecular targets. More importantly, the overall prognosis of breast cancer remains unsatisfactory owing to individual heterogeneity (3). Therefore, developing accurate strategies for predicting prognosis is necessary for improving clinical management. Currently, machine learning and bioinformatics are widely utilized as the mothodology to exploit robust models focusing on diverse end events (4–8). Compared with single indicators such as a clinicopathological parameter or the expression of a single gene, an integrated signature comprising several pivotal features appears to be a more robust tool for predicting prognosis. Therefore, exploitation and application of a valuable signature to predict the prognosis of patients with breast cancer may help in clinical decision making, prioritizing survival improvement.

The degradome is a repertoire of all proteases expressed in an organism, with over 550 protease-coding genes being identified in the human genome (9). It is preliminarily divided into five clusters according to catalytic sites: aspartyl proteases, cysteine proteases, serine proteases, threonine proteases and metalloproteases (9). The degradome provides a different insight into the functional dysregulation of cancer cells caused by protein/peptide degradation and modification, which may help to elucidate oncogenic mechanisms and identify potential drug targets. Structural alteration or aberrant expression of degradome-related genes (DRGs) has been associated with diverse human diseases, including neurodegenerative disorders (10), cardiovascular diseases (11), musculoskeletal diseases (12), bowel diseases (13) and, particularly, malignancy. Numerous DRGs have been associated with the phenotype of breast cancer. DNPEP (an aspartyl protease) is sponged by PAK5 in breast cancer cells, and its overexpression attenuates cell proliferation and invasion *in vitro* and suppresses tumour growth and metastasis *in vivo* (14). USP4 (a cysteine protease) has been identified as the downstream target of DNPEP in the PAK5/DNPEP/USP4 regulatory axis in breast cancer. High USP4 expression is associated with the poor prognosis of patients with breast cancer (14). The expression of TMPRSS13 (a transmembrane serine protease) is elevated in IDC tissues. Silencing TMPRSS13 can significantly suppress breast cancer progression both *in vitro* and *in vivo* by decreasing proliferation, enhancing apoptosis and inhibiting invasion, resulting in the inhibition of overall tumour burden and deficiency of detectable tumour growth (15). Additionally, TMPRSS13 knockdown sensitizes aggressive TNBC cells to chemotherapy agents *in vitro*. PRSS8 (a serine protease) accumulation mediated by TMPRSS13 knockdown is a potential tumour-suppressive mechanism (15). TASP1 (a threonine protease) plays an essential role in both normal mammary gland development and breast cancer progression (16). TASP1 knockdown reduces the expression of cyclins E and A *in vivo*, thereby blocking carcinogenesis. Mixed-lineage leukaemia has been identified as a major substrate of TASP1 and is required for the development of HER2-positive breast cancer *in vitro* (16). Matrix metalloproteases (MMPs) are well-known members of metalloproteases. MMPs may promote breast cancer progression by remodeling the tumor microenvironment (17). Therefore, constructing a comprehensive prognostic signature based on DRGs may help to understand their prognostic value in breast cancer in a broader way.

In this study, we developed and validated a prognostic signature based on DRGs. Investigations of the degradome signature with respect to clinical association, functional characterization, mutation landscape, immune infiltration and immune checkpoint expression of were orchestrated. Additionally, the clinical utility of the signature in predicting the prognosis of patients undergoing different therapies was analyzed, and potential drugs for chemotherapy and molecular targeted therapy were also implied in different risk groups. *In vitro* experiments further confirmed the molecular functions of two DGRs (ABHD12 and USP41).

## 2 Materials and methods

### 2.1 Data acquisition and pre-processing

The transcriptome data and clinical information of patients with breast invasive carcinoma (BRCA) were downloaded from TCGA (http://cancergenome.nih.gov/), GEO (GSE96058 dataset) (https://www.ncbi.nlm.nih.gov/geo) and Molecular Taxonomy of Breast Cancer International Consortium (METABRIC) databases. Male BRCA samples and samples without survival information were excluded. Eventually, 1069 BRCA and 113 normal samples from TCGA, 3273 BRCA samples from GSE96058 and 1904 BRCA samples from METABRIC were included.

### 2.2 Identification of degradome-related genes

DRGs were selected from The Mammalian Degradome Database (degradome.uniovi.es/dindex.html) (18). The DESeq2 R package was used to screen for differentially expressed genes (DEGs) between BRCA and normal samples in TCGA cohort. Genes with |Log_2_FC| value > 1 and *P*-value < 0.05 were considered as DEGs. The survival R package was used to screen for prognosis-related genes (PRGs) significantly correlate with overall survival (OS) in TCGA cohort. These DRGs, DEGs and PRGs were intersected, and 22 overlapping DRGs were selected for subsequent analysis. The expression pattern of the 22 DRGs and the correlation among them were analyzed. The clusterProfiler and org.Hs.eg.db R packages were used for Gene Ontology (GO) and Kyoto Encyclopedia of Genes and Genomes (KEGG) functional enrichment analyses of the 22 DRGs. Besides, ConsensusClusterPlus R package was used to perform consensus clustering to verify the consistence of the 22 DGRs by means of dissecting subtypes in TCGA cohort.

### 2.3 Construction of degradome-based prognostic signature

TCGA cohort was used as the training cohort to construct a degradome-based prognostic signature. Univariate and multivariate Cox regression analyses were performed to identify independent prognostic predictors from the 22 DRGs. Thereafter, LASSO regression analysis was employed to construct a prognostic signature based on the 22 DRGs. The risk score was calculated as follows: Risk score = ∑(C_i_*E_i_). In the equation, *i* represents a certain DRG, *C* represents the coefficient of the DRG and *E* represents the expression level of the DRG. Patients were divided into the low- and high-risk groups according to the median risk score. The survminer and survival R packages were used to compare patient survival between the two groups. The timeROC R package was used to plot receiver operating characteristic (ROC) curves to assess the predictive ability of the degradome signature.

### 2.4 Validation of the degradome signature

The GSE96058 dataset was used for external validation. Three cohorts from TCGA were selected for internal validation: pathological-stage-III, ER-positive and HER2-positive cohorts. The degradome signature was tested in each cohort. Kaplan–Meier (K-M) survival curves and ROC curves were plotted for each cohort. Principal component analysis (PCA) was further used to differentiate the low- and high-risk groups in both TCGA and GSE96058 cohorts. In addition, we extracted 18 clinical subgroups from TCGA cohort to clarify the applicability of the degradome signature in a more broader way.

### 2.5 Clinicopathological differences between the two risk groups

The clinicopathological parameters (11 parameters) and complete response of patients undergoing different therapies (chemotherapy, endocrinotherapy and radiotherapy) were compared between the low- and high-risk groups. K-M survival curves were plotted to compare disease-specific survival (DSS), disease-free interval (DFI) and progression-free interval (PFI) between the low- and high-risk groups.

### 2.6 Development of degradome-related clinicopathological nomogram

Univariate and multivariate Cox regression analyses were performed based on 11 clinicopathological parameters and risk score. Characteristics with *P*-value < 0.05 from the multivariate Cox regression analysis were further used to develop a nomogram to predict OS. The predictive accuracy of the nomogram was verified based on the concordance index (C-index) and calibration curves. Besides, decision curve analysis (DCA) was performed to assess the advantage in clinical benefit of the nomogram compared with traditional pathological stage.

### 2.7 Functional characterisation of DEGs in the two risk groups

DEGs between the low- and high-risk groups were identified and named new DEGs (nDEGs). NetworkAnalyst (http://www.networkanalyst.ca) was employed to build a protein–protein interaction (PPI) network based on nDEGs. The nDEGs were functionally characterized *via* GO and KEGG analyses based on a pre-determined |log_2_FC| threshold. Gene set enrichment analysis (GSEA) was performed to identify the significantly enriched functional pathways in the two risk groups. In addition, the DRGs and nDEGs were intersected to compare the degradome expression pattern between the low- and high-risk groups.

### 2.8 Mutation landscapes of the two risk groups

The Breast Invasive Carcinoma dataset (TCGA, PanCancer Atlas 1084 samples) in the cBioPortal for Cancer Genomics database (http://www.cbioportal.org) was used for subsequent analysis. The mutation landscape of the whole cohort, low-risk group and high-risk group was respectively extracted. The top 10 most frequently altered genes and DRGs in the two risk groups were determined. Differences in the fraction of genome altered, mutation counts, microsatellite instability (MSI) and tumor mutation burden (TMB) were analyzed between the low- and high-risk groups. Samples were divided into the low- and high-TMB groups according to the median TMB value. Subsequently, differences in survival were analyzed between the low- and high-TMB groups with or without the consideration of risk score. Additionally, the correlation between risk score and TMB was analyzed.

### 2.9 Differences in immune infiltration and immune checkpoint expression between the two risk groups

The GEPIA2021 database (gepia2021.cancer-pku.cn) was used to examine the correlation among the infiltration of 22 types of immune cells in the tumour microenvironment (TME) of breast cancer. The CIBERSORT algorithm was used to evaluate the infiltration levels of 22 types of immune cells. The correlation between risk score and 22 types of immune infiltrating cells was analyzed, and the infiltration levels were also compared between the low- and high-risk groups. Furthermore, the expression pattern of 47 immune checkpoints was compared between the two risk groups to assess the potential value of the degradome signature in immunotherapy.

### 2.10 Prognostic prediction of patients undergoing different therapies

The survival of patients undergoing different therapies was compared between the low- and high-risk groups in TCGA and METABRIC cohorts. Potential drugs that may result in complete response to chemotherapy were identified in the two risk groups. Additionally, the expression pattern of 21 potential molecular targets from the PI3K/AKT/mTOR signaling pathway, CDK family and PARP family was examined to ascertain targets for molecular targeted therapy in different risk groups.

### 2.11 Drug sensitivity analysis

With pRRophetic R package, we processed wide drug screening based on GDSC database (https://www.sanger.ac.uk/tool/gdsc-genomics-drug-sensitivity-cancer) to ascertain the drugs that the two risk groups may sensitively respond to.

### 2.12 Cell culture and siRNA transfection

Two human breast cancer cell lines MCF-7 and MDA-MB-435S were purchased from Wuhan Procell Life Science and Technology Co., Ltd. (Wuhan, China) to form parallel contrast. Cells were cultured in RPMI-1640 (Gibco-BRL) supplemented with 10% foetal bovine serum (Bioserum), 100-U/mL penicillin G and 100-μg/mL streptomycin. siRNAs targeting ABHD12 and USP41 were purchased from GeneChem (Genechem Co., Ltd, Shanghai, China). The siRNAs were transfected into MCF-7 and MDA-MB-435S cells following the recommended guidelines. For each gene (ABHD12 or USP41), a total of three groups were formed: normal control (NC), siRNA1 and siRNA2 groups. The sequence of siRNAs were provided in **Supplementary Material**.

### 2.13 Western blotting

MCF-7 cells and MDA-MB-435S cells were lysed in radioimmunoprecipitation assay (RIPA) buffer (Zhonghuihecai, Xi’an, China) and pelleted *via* centrifugation at 4°C for 15 min, and the supernatant was discarded. Subsequently, 1/5 sodium dodecyl sulfate–polyacrylamide gel loading buffer (5×; Beyotime, Shanghai, China) was added, and the sample was heated in a 100°C metal bath for 10 min. The extracted proteins were separated on a 15% sodium dodecyl sulfate–polyacrylamide gel and transferred to a 0.22-mm polyvinylidene fluoride (PVDF) membrane (Millipore, USA). The membrane was blocked with 5% skimmed milk for approximately 2 h and incubated with specific antibodies: ABHD12 (Cat. No.: EPR13683, 1:100,000, Abcam), USP41 (Cat. No.: PA5-71281, 1:100,000, Invitrogen) and β-actin (Cat. No.: Ab6276, 1:100,000, Abcam). The protein bands were visualised using a chemiluminescent kit (Vazyme, Nanjing, China).

### 2.14 Cell phenotype assays

Colony formation and CCK8 assays were performed to assess the proliferation of breast cancer cells. Cells from different groups were digested and inoculated in 6-well plates (Jet Biofilter Co., Ltd., Guangzhou, China), with 1,000 cells per well. The medium was changed every 3 days, and the cells were cultured for 10–14 days. After visible colonies were formed, they were immobilised with 4% paraformaldehyde and stained with crystalline violet (Solarbio Life Sciences, China). CCK8 assay (Dojindo, Tokyo, Japan) was performed according to the manufacturer’s instructions. The absorbance was measured at 450 nm using a microplate reader. Wound healing and transwell assays were performed to assess the invasive and migratory abilities of breast cancer cells. Cells from different groups were digested and inoculated in 6-well plates. After the cells had reached 95% confluence, a straight scratch was made on the surface of each well with the tip of a 100-mL sterile pipette. The wound area was photographed using an inverted microscope (Nikon DS-RI2, Japan) at 100× magnification at 0, 12 and 24 h. For transwell assay, 800 mL of a medium containing 10% foetal bovine serum (Corning, USA) was added to the lower chamber, and 200 mL of a serum-free medium containing 20,000 cells was added to the upper Matrigel-coated chamber. After 24 h of incubation, cells that had crossed the membrane were fixed with 4% paraformaldehyde, washed with phosphate-buffered saline, stained with crystal violet and photographed using an inverted microscope at 200× magnification.

### 2.15 Statistical analysis

All statistical analyses were performed using R 4.0.3. K-M survival curves were compared *via* Cox regression analysis. The Wilcoxon rank sum test was used to compare the gene expression between groups, and the chi-square test was used to compare the differences in clinicopathological parameters between risk groups. Spearman correlation coefficients were evaluated for correlation analysis. |*r*| value > 0.1 were considered relevant, and *P*-value < 0.05 were considered statistically significant. ‘*’ indicates *P*-value < 0.05, ‘**’ indicates *P*-value < 0.01 and ‘***’ indicates *P*-value < 0.001 throughout this study.

## 3 Results

### 3.1 22 DRGs correlated with prognosis were demarcated

**Figure 1:**
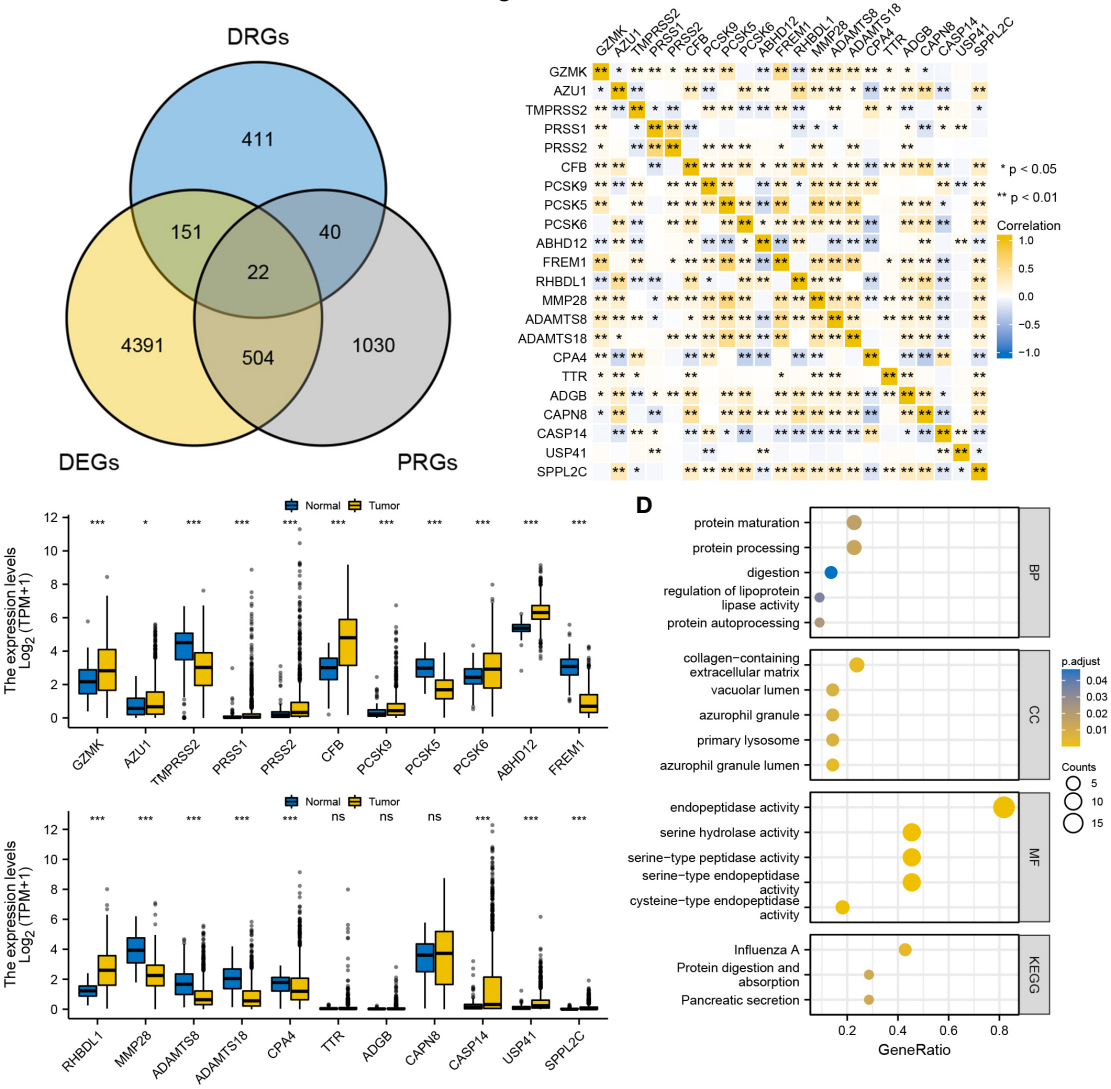
Primary identification and verification of DRGs in BRCA. **(A)** Intersection of DRGs, DEGs and PRGs. **(B)** Expression pattern of the 22 DRGs between BRCA and the normal. **(C)** Correlation among the expression of the 22 DRGs in BRCA. **(D)** GO/KEGG functional enrichment analyses of the 22 DRGs. ‘***’ indicates *P*-value < 0.001. "ns" represents non-significant.

A total of 625 DRGs were selected from The Mammalian Degradome Database, including 24 aspartyl proteases, 169 cysteine proteases, 201 serine proteases, 28 threonine proteases and 203 metalloproteases. In the meanwhile, a total of 5068 DEGs and 1596 PRGs were identified. These three gene clusters were intersected (**Figure 1A**), and the expression patterns of the 22 overlapping DRGs were verified (**Figure 1B**). Furthermore, the correlation among the expression of the 22 DRGs was examined (**Figure 1C**). The expression of most DRGs was positively correlated. Functional enrichment analysis confirmed that the 22 DRGs are associated with protein degradation and processing (**Figure 1D**). Additionally, two subtypes (BRCA subtype 1 and BRCA subtype 2) were concisely divided *via* consensus clustering, verifying the favourable consistence of the 22 DRGs (**Supplementary Figure S1**).

### 3.2 A 10-gene degradome-based signature was constructed for predicting prognosis

**Figure 2:**
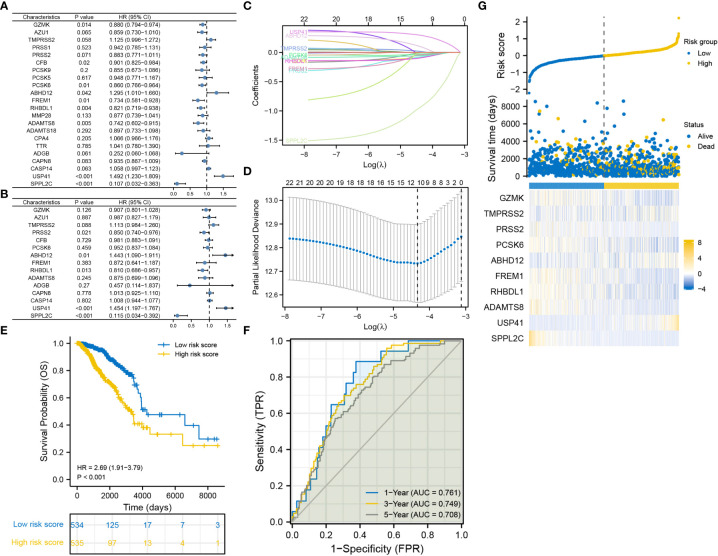
Construction of the degradome signature *via* LASSO regression analysis. **(A)** Univariate Cox regression analysis. **(B)** Multivariate Cox regression analysis. **(C, D)** LASSO regression analysis. **(E)** K-M curve for comparing OS between the low- and high-risk groups. **(F)** ROC curves of the degradome signature at 1-, 3- and 5-year. **(G)** Distribution of risk scores and survival time (days) between the low- and high-risk groups.

A total of 5 DRGs were identified as independent prognostic predictors in BRCA (**Figures 2A, B**). PRSS2, SPPL2C and RHBDL1 were identified as protective factors, whereas USP41 and ABHD12 were identified as risky factors. A 10-gene signature was constructed *via* LASSO regression analysis (**Figures 2C, D**), and the risk score was calculated as follows: (-0.0776)*GZMK expression + (-0.0153)*TMPRSS2 expression + (-0.0742)*PRSS2 expression + (-0.0251)*PCSK6 expression + (0.0913)*ABHD12 expression + (-0.0405)*FREM1 expression + (-0.0902)*RHBDL1 expression + (-0.0540)*ADAMTS8 expression + (0.2896)*USP41 expression + (-0.9196)*SPPL2C expression. K-M curves revealed that OS was worse in the high-risk group than in the low-risk group (**Figure 2E**). The area under the ROC curve (AUC) was 0.761, 0.749 and 0.708 at 1-, 3- and 5-year, respectively (**Figure 2F**). The distribution of risk scores and survival time (days) were also displayed between the low- and high-risk groups (**Figure 2G**).

### 3.3 Degradome signature was fairly validated in internal and external cohorts

**Figure 3:**
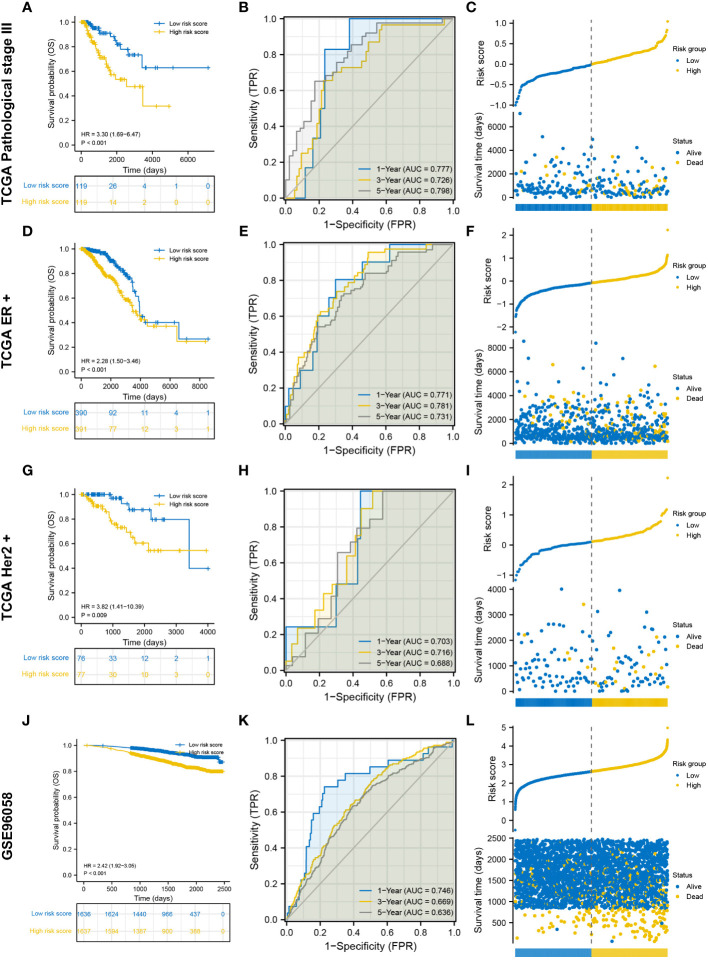
Internal and external validation of the degradome signature. **(A–C)** Internal validation based on TCGA pathological stage III cohort. **(D–F)** Internal validation based on TCGA ER-positive cohort. **(G–I)** Internal validation based on TCGA HER2-positive cohort. **(J–L)** External validation based on GSE96058 dataset.

Three TCGA cohorts were used for internal validation: pathological stage III, ER-positive and HER2-positive cohorts. Survival was adequately distinguished between the low- and high-risk groups in the three cohorts, with patients in the high-risk group having poorer outcomes (**Figures 3A, D, G**). In the pathological stage III cohort, the AUCs for predicting survival probability at 1-, 3- and 5-year were 0.777, 0.726 and 0.798, respectively (**Figure 3B**). In the ER-positive cohort, the AUCs for predicting survival probability at 1-, 3- and 5-year were 0.771, 0.781 and 0.731, respectively (**Figure 3E**). In the HER2-positive cohort, the AUCs for predicting survival probability at 1-, 3- and 5-year were 0.703, 0.716 and 0.688, respectively (**Figure 3H**). The GSE96058 dataset was used for external validation. Patients in the high-risk group harbored worse prognosis (**Figure 3J**). The AUCs for predicting survival probability at 1-, 3- and 5-year were 0.746, 0.669 and 0.636, respectively (**Figure 3K**). The distribution of patients with risk score and survival time in the three TCGA cohorts and GSE96058 cohort were demonstrated, respectively (**Figures 3C, F, I, L**). The distinct risk grouping in both TCGA and GSE96058 cohorts was confirmed by PCA (**Supplementary Figure S2**). Besides, the AUC stands for predictive capability of risk score was prior to other clinicopathological characteristics but age in both TCGA and GSE96058 cohorts (**Supplementary Figure S3**). Subgroup analysis further determined the robust efficacy of the degradome signature in discriminating prognosis regardless of multiple clinicopathological features (**Supplementary Figure S4**). Altogether, these results verified the favourable applicability of the degradome signature.

### 3.4 Degradome-related clinicopathological nomogram showed moderate predictive capability

**Figure 4:**
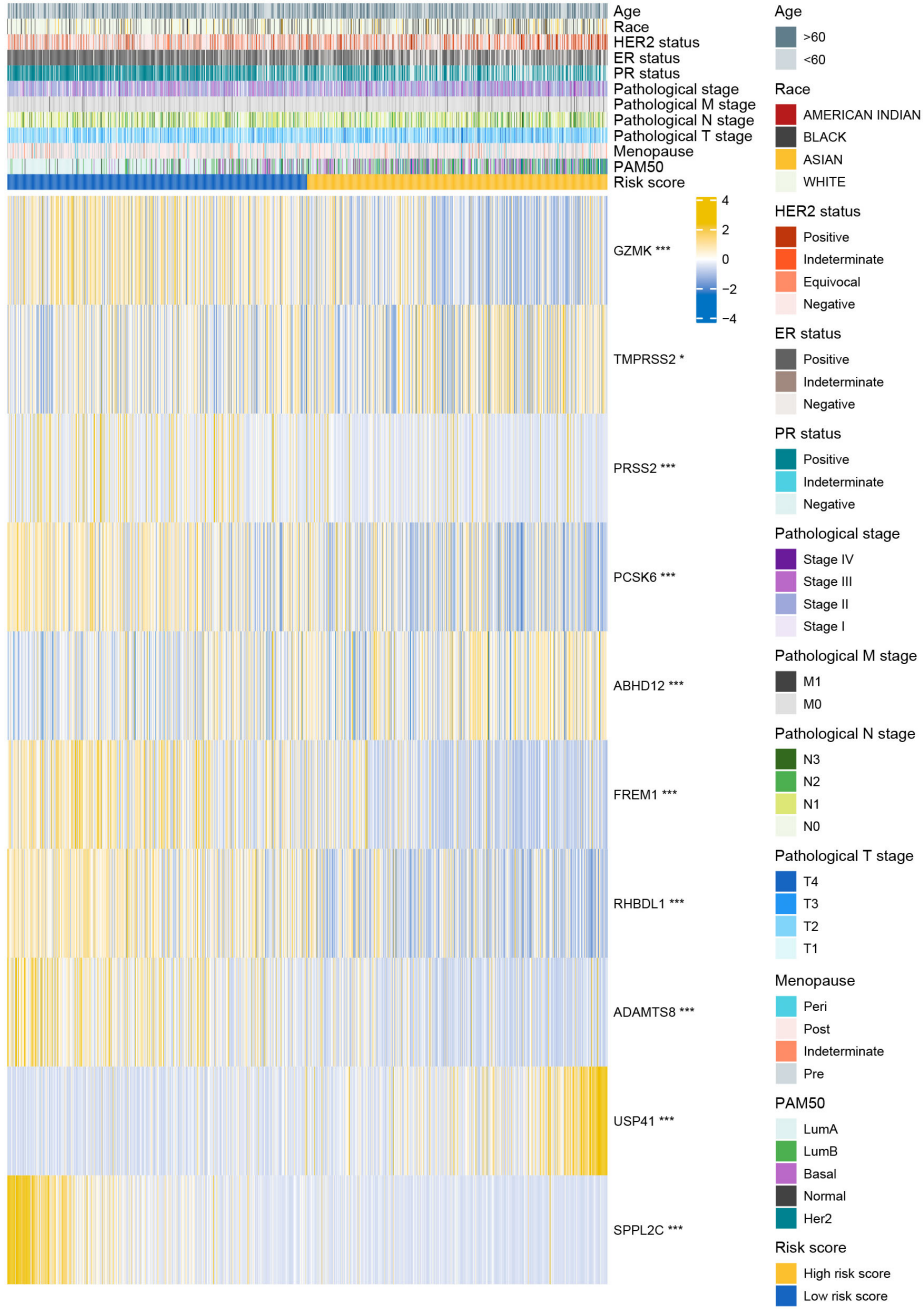
Distribution of clinicopathological characteristics in the low- and high-risk groups.

**Figure 5:**
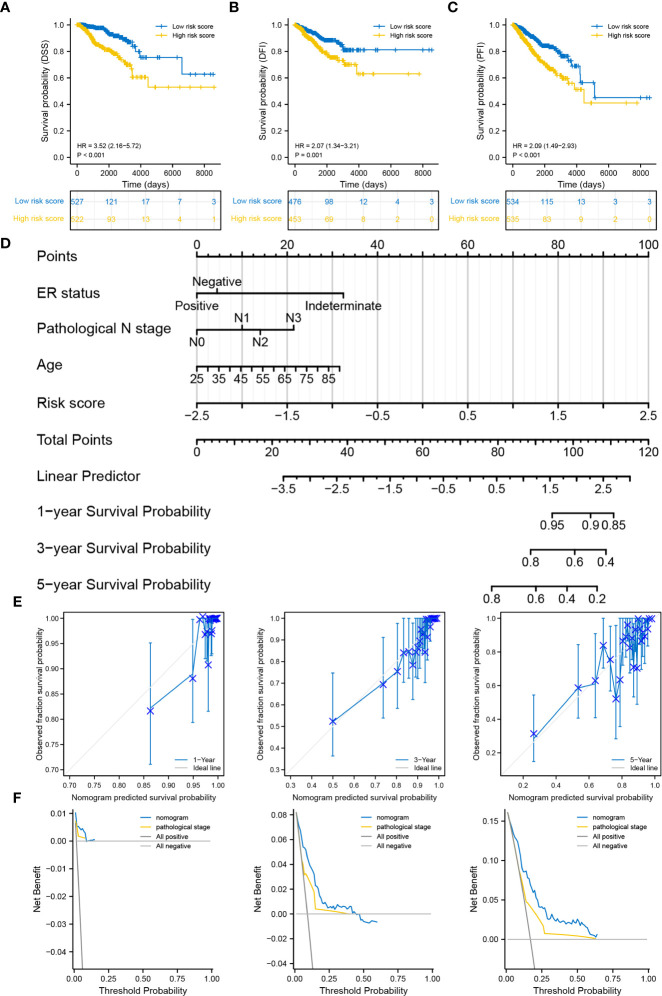
Correlation between the degradome signature and clinicopathological characteristics. **(A–C)** Survival differences in DSS, DFI and PFI between the low- and high-risk groups. **(D)** Prognostic nomogram based on clinicopathological characteristics and risk score. **(E)** Calibration curves of the nomogram at 1-, 3- and 5-year. **(F)** DCA of the nomogram at 1-, 3- and 5-year.

The distributions of 11 clinicopathological parameters were displayed between the low- and high-risk groups, with integration of the expression of 10 DRGs (**Figure 4**). These 10 DRGs were significantly differentially expressed between the low- and high-risk groups. On comparing the differences in clinicopathological characteristics between the two risk groups (**Supplementary Table S1**), the high-risk group was found to have more patients with T4 stage, ER-negative, PR-negative and HER2-positive breast cancers and more patients in the post-menopausal status. Regarding the PAM50 subtype, the high-risk group had more patients with the Her2, basal and luminal B subtypes, whereas the low-risk group had more patients with luminal A subtype. In addition, the proportion of patients with complete response to chemotherapy was higher in the low-risk group than in the high-risk group. However, no significant differences in complete response to endocrinotherapy and radiotherapy were observed between the two risk groups. Moreover, patients in the high-risk group had worse DSS, DFI and PFI (**Figures 5A-C**).

Age, ER status (negative), N stage (N3) and risk score were deciphered as independent prognostic predictors of BRCA (**Supplementary Table S2**). The risk score was also identified as an independent prognostic predictor in the four validation cohorts (**Supplementary Figure S5**). The four factors were subsequently used to develop a nomogram for predicting OS at 1-, 3- and 5-year (**Figure 5D**). The C-index was 0.769 (0.747-0.790), demonstrating the robust predictive ability of the nomogram. The risk score had the strongest effect on OS according to its wide point contribution. The calibration curves were close to the ideal line, suggesting excellent predictive efficacy of the nomogram (**Figure 5E**). Additionally, DCA revealed that the nomogram has more satisfactory clinical decision-making advantages at 1-, 3- and 5-year comparing with the traditional pathological stage (**Figure 5F**).

### 3.5 TLR signaling regulation and cell cycle-promoting activities were significantly upregulated in the high-risk group

**Figure 6:**
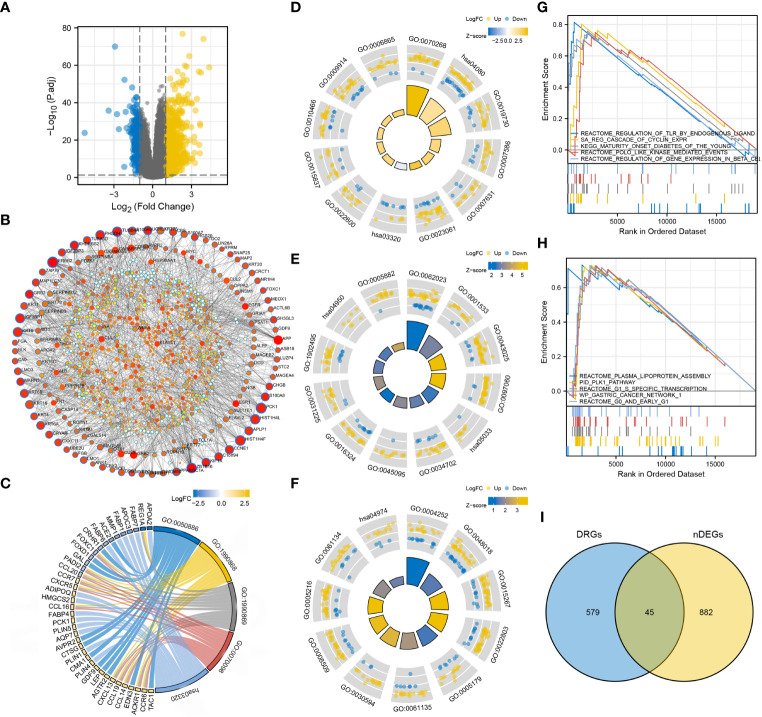
Functional characterization in the two risk groups. **(A)** DEGs between the low- and high-risk groups. **(B)** Tissue-specific PPI network of the DEGs. Dots with blue borders represent nDEGs. Dots with a larger size and stronger colour intensity indicate nDEGs that play a more important role in the PPI network. **(C)** GO/KEGG biological activities that are significantly enriched in the low-risk group. **(D–F)** GO/KEGG biological activities that are significantly enriched in the high-risk group. **(G, H)** GSEA in the high-risk group. **(I)** Identification of DR-nDEGs.

**Figure 7:**
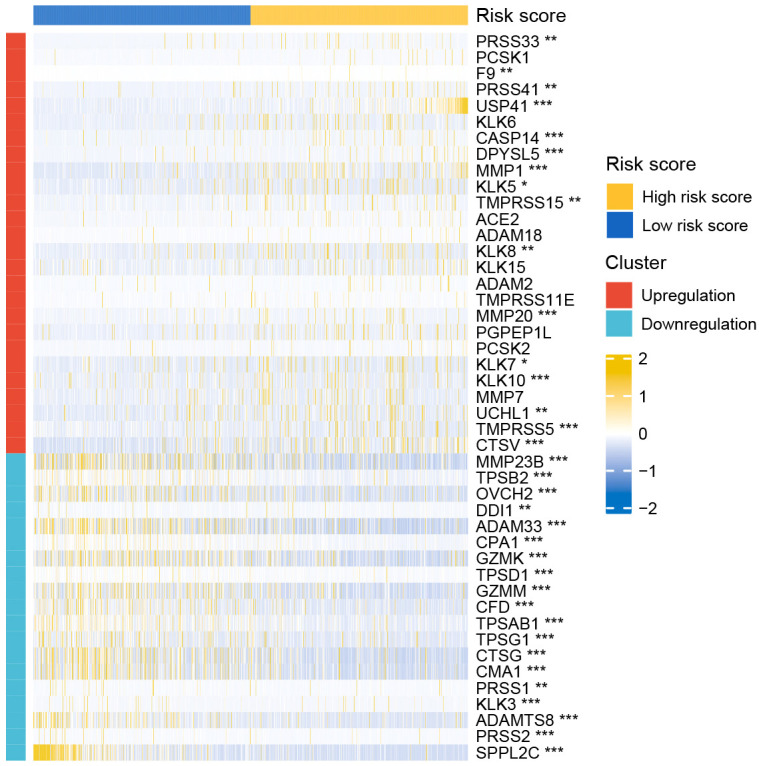
Expression pattern of DRGs between the low- and high-risk groups.

nDEGs between the two risk groups were identified and used to construct a tissue-specific PPI network (**Figures 6A, B**). GO/KEGG analysis revealed that four biological activities mainly associate with chemokine responses were significantly upregulated in the low-risk group (z score > 1) (**Figure 6C**). A total of 30 GO terms and 5 KEGG terms were significantly upregulated in the high-risk group (z score > 1) (**Figures 6D–F**). These terms included cornification, antimicrobial humoral response, digestion, collagen-containing extracellular matrix, cornified envelope, neuronal cell body, serine-type endopeptidase activity, receptor-ligand activity, channel activity, neuroactive ligand-receptor interaction, PPAR signaling pathway and nicotine addiction, etc. The detailed results of functional annotation are provided in **Supplementary Table S3**. Subsequently, GSEA revealed the biological activities and signaling pathways enriched in the high-risk group (**Figures 6G, H**). These activities and pathways were mainly summarized as the following two aspects: TLR signalling regulation (regulation of TLRs by endogenous ligands) and cell cycle-promoting activities (REG cascade of cyclin EXPR, polo-like kinase mediated events, PLK1 pathway, G0 and early G1 and G1 specific transcription). However, no significant GSEA results were observed in the low-risk group. Furthermore, a total of 45 degradome-related nDEGs (DR-nDEGs) were identified after the intersection of DRGs and nDEGs (**Figure 6I**). The expressions of the 45 DR-nDEGs were compared between the low- and high-risk groups, suggesting the distinct degradome pattern in different risk groups (**Figure 7**).

### 3.6 Different risk groups had distinct mutation characteristics

**Figure 8:**
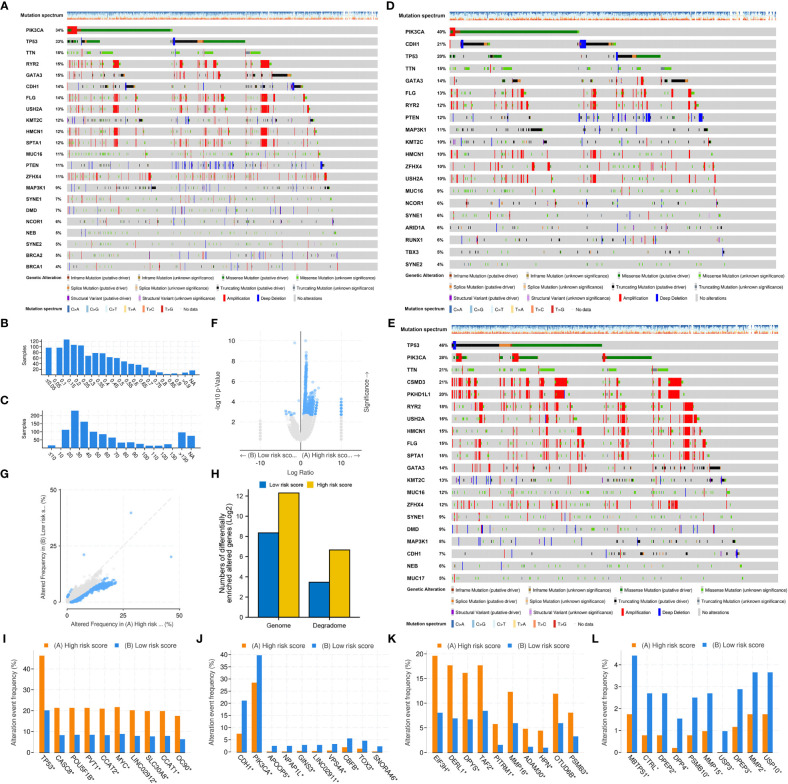
Mutation differences between the low- and high-risk groups. **(A)** Mutation landscape of the whole TCGA-BRCA cohort. **(B)** Fraction of genome altered. **(C)** Gene mutation count. **(D)** Mutation landscape of the low-risk group. **(E)** Mutation landscape of the high-risk group. **(F, G)** Significantly altered genes between the low- and high-risk groups. **(H)** Genes with significantly different mutation frequencies in the entire genome and degradome in the two risk groups. **(I, J)** The top 10 genes with significantly different mutation frequencies in the high- and low-risk groups. **(K, L)** The top 10 DRGs with significantly different mutation frequencies in the high- and low-risk groups.

The top 20 most frequently altered genes in BRCA, plus the two putative genes, BRCA1 and BRCA2, were examined (**Figure 8A**). The mutation frequency of PIK3CA (34%) and TP53 (33%) was the highest, with the most common mutation type being missense mutation. The fraction of genome altered was less than 25% in most patients (**Figure 8B**). The gene mutation count of most patients ranged from 10 to 40 (**Figure 8C**). Furthermore, the gene mutation landscapes of the low- and high-risk groups were determined, respectively (**Figures 8D, E**). PIK3CA (40%) and TP53 (46%) were the most frequently altered genes in the low- and high-risk groups, respectively. This finding indicated the different gene-driven oncogenesis in patients with different risk statuses. Subsequently, significantly altered genes between the two risk groups were identified (**Figures 8F, G**). The frequency of significant gene mutations was higher in the high-risk group. Genes with significantly different mutation frequencies between the two risk groups were further counted (**Figure 8H**). For both the entire genome and degradome, the number of significantly mutated genes was higher in the high-risk group. The top 10 significantly mutated genes in the entire genome and degradome in the two risk groups were respectively displayed (**Figures 8I–L**).

**Figure 9:**
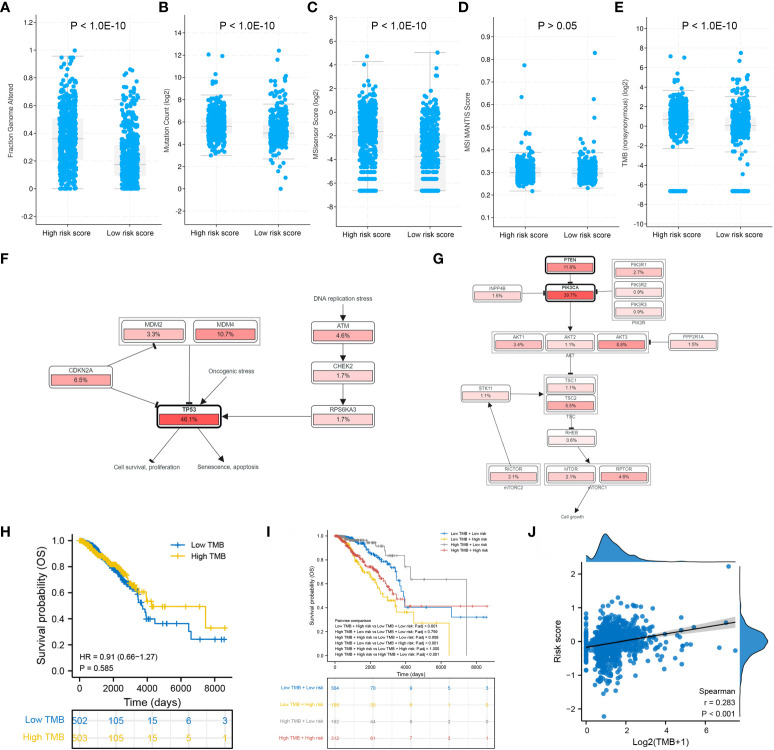
Mutation differences between the low- and high-risk groups. Differences in **(A)** fraction of genome altered, **(B)** gene mutation count, **(C)** MSIsensor score, **(D)** MSI MANTIS score and **(E)** TMB between the two risk groups. Dominant signaling pathways in the **(F)** high-risk group and **(G)** low-risk group. **(H)** Survival analysis between the low- and high-TMB groups. **(I)** Survival analysis integrating the risk score and TMB. **(J)** Correlation between the risk score and TMB.

Patients in the high-risk group had a significantly higher fraction of altered genome, more gene mutation counts, higher MSIsensor scores and higher TMB than patients in the low-risk group (**Figures 9A–E**). The dominant oncogenic pathway was checked out to be the TP53 signaling pathway in the high-risk group and the PI3K/AKT/mTOR signaling pathway in the low-risk group (**Figures 9F, G**). These results are consistent with those of gene mutation landscapes. However, TMB was found to have no prognostic value in BRCA (**Figure 9H**). Survival analysis integrating risk score and TMB revealed that patients with low TMB and high risk score had the worst prognosis, whereas those with high TMB and low risk score had the best prognosis (**Figure 9I**). More importantly, the risk score was significantly positively correlated with TMB (*r* = 0.283, *P* < 0.001) (**Figure 9J**).

### 3.7 Degradome signature was correlated with immune infiltration and immune checkpoint expression

**Figure 10:**
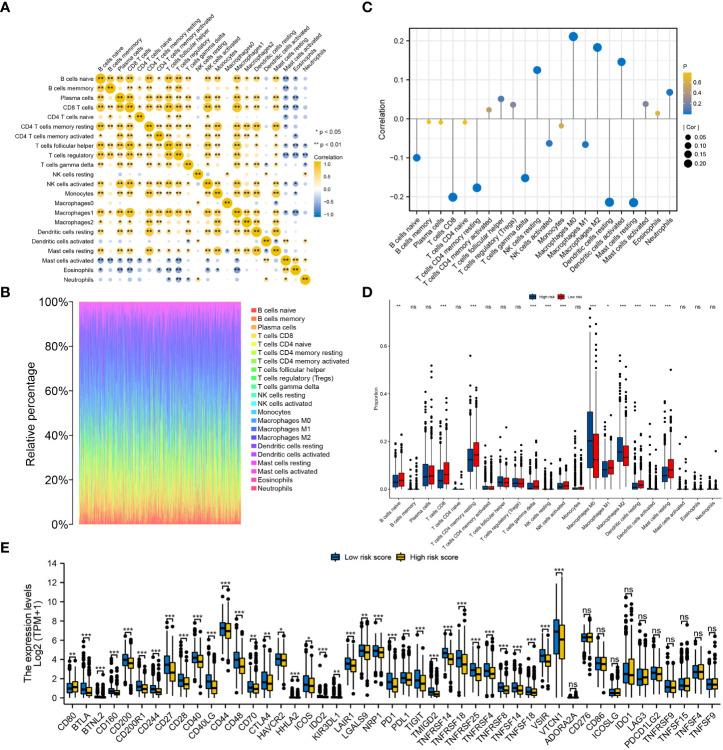
Evaluation of the infiltration levels of immune cells and the expression pattern of immune checkpoints between the low- and high-risk groups. **(A)** Correlation among the infiltration levels of 22 types of immune cells in BRCA. **(B)** Infiltration levels of immune cells in each BRCA sample. **(C)** Correlation between the risk score and infiltration levels of the 22 types of immune cells. **(D)** Differences in the infiltration levels of the 22 types of immune cells between the two risk groups. **(E)** Differential expression of 47 immune checkpoints between the low- and high-risk groups. ‘***’ indicates *P*-value < 0.001. "ns" represents non-significant.

The infiltration levels of a majority of immune cells were positively correlated with each other in BRCA (**Figure 10A**). The infiltration levels of 22 types of immune cells were evaluated in each BRCA sample (**Figure 10B**). The risk score was significantly positively correlated with the infiltration levels of resting NK cells, M0 and M2 macrophages, activated dendritic cells and neutrophils and significantly negatively correlated with the infiltration levels of naive B cells, CD8 T cells, resting memory CD4 T cells, gamma-delta T cells, activated NK cells, M1 macrophages, resting dendritic cells and resting mast cells (**Figure 10C**). Furthermore, the infiltration levels of resting NK cells, macrophages M0, macrophages M2 and activated dendritic cells were significantly higher in the high-risk group, whereas those of naive B cells, CD8 T cells, resting memory CD4 T cells, gamma-delta T cells, activated NK cells, M1 macrophages, resting dendritic cells and resting mast cells were significantly higher in the low-risk group (**Figure 10D**). Only CD80 was significantly upregulated in the high-risk group, while the other 35 immune checkpoints were significantly upregulated in the low-risk group, including CTLA4, PD1 and PDL1 (**Figure 10E**).

### 3.8 Degradome signature efficiently predicted the prognosis of patients undergoing endocrinotherapy or radiotherapy

**Figure 11:**
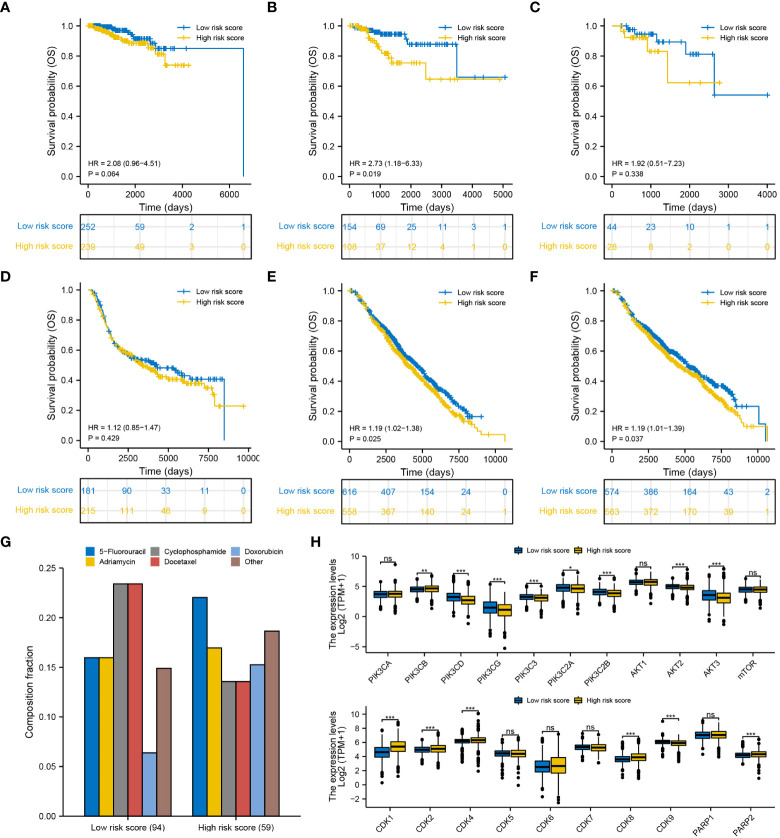
Efficiency of the degradome signature in predicting the prognosis of patients with BRCA undergoing different therapies. Differences in the survival of patients undergoing **(A)** chemotherapy, **(B)** endocrinotherapy and **(C)** radiotherapy between the low- and high-risk groups in TCGA cohort. Differences in the survival of patients undergoing **(D)** chemotherapy, **(E)** endocrinotherapy and **(F)** radiotherapy between the low- and high-risk groups in the METABRIC cohort. **(G)** Proportion of patients with complete response to the first-round chemotherapy and the corresponding drug agents in the two risk groups. **(H)** Expression pattern of 21 potential molecular targets between the low- and high-risk groups.

The efficiency of the degradome signature in predicting the prognosis of patients undergoing different therapies was examined in TCGA and METABRIC cohorts, respectively. The prognosis of patients undergoing traditional chemotherapy was not significantly distinguished between the low- and high-risk groups in both TCGA and METABRIC cohorts (**Figures 11A, D**). The prognosis of patients undergoing endocrinotherapy was worse in the high-risk group than in the low-risk group in both TCGA and METABRIC cohorts (**Figures 11B, E**). The prognosis of patients undergoing radiotherapy was poorer in the high-risk group than in the low-risk group in the METABRIC cohort (**Figure 11F**). However, there was no difference in the prognosis of patients undergoing radiotherapy between the low- and high-risk groups in TCGA cohort, possibly owing to limited samples (**Figure 11C**).

We next summarized the TCGA-BRCA samples that gain complete response after first-round traditional chemotherapy and the corresponding drug agents (**Figure 11G**). A total of 94 and 59 patients exhibited complete response to the first-round traditional chemotherapy in the low- and high-risk groups, respectively. Cyclophosphamide and docetaxel were found to be more beneficial for patients in the low-risk group, while 5-fluorouracil may be more proper for patients in the high-risk group to gain complete response. Furthermore, the expression pattern of 21 molecules in the PI3K/AKT/mTOR signaling pathway, CDK family and PARP family were investigated between different risk groups (**Figure 11H**). Most targets from the PI3K/AKT/mTOR signaling pathway were significantly upregulated in the low-risk group, whereas most targets from the CDK family and PARP family were significantly upregulated in the high-risk group. These targets may potentially serve for molecular targeted therapy in BRCA. Interestingly, drug sensitivity analysis with IC50 further suggested that docetaxel, epirubicin and inhibitors of PI3K/AKT/mTOR signaling pathway (afuresertib, buparlisib, ipatasertib and dactolisib) are more beneficial to patients in the low-risk group, whereas tyrosine kinase inhibitors (ibrutinib, lapatinib and sapitinib) may better benefit patients in the high-risk group (**Supplementary Figure S6**).

### 3.9 Sponging ABHD12 and USP41 significantly inhibited the proliferation, invasion and migration of breast cancer cells

**Figure 12:**
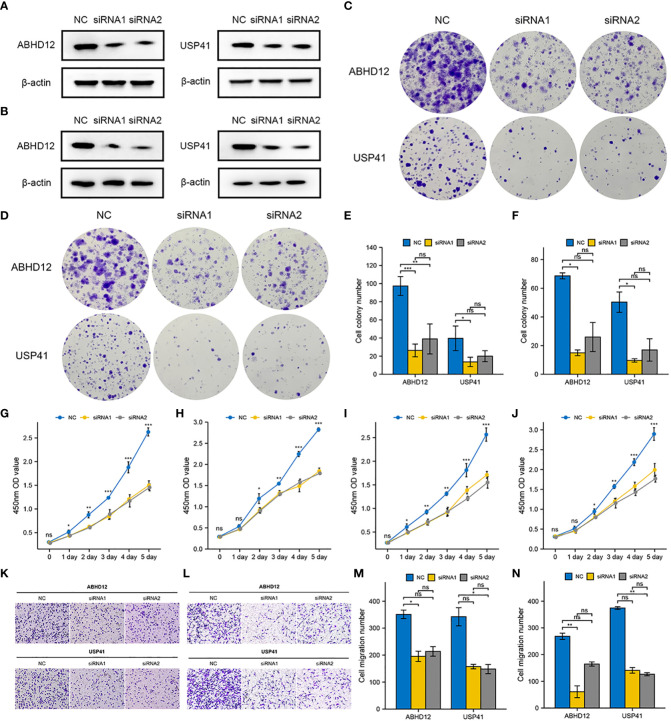
Effects of ABHD12 and USP41 on cell proliferation and migration. **(A)** Evaluation of silencing of ABHD12 and USP41 *via* western blotting in MCF-7 cells. **(B)** Evaluation of silencing of ABHD12 and USP41 *via* western blotting in MDA-MB-435S cells. **(C, E)** Colony formation assay in MCF-7 cells. **(D, F)** Colony formation assay in MDA-MB-435S cells. **(G, H)** CCK8 assay in MCF-7 cells. **(I, J)** CCK8 assay in MDA-MB-435S cells. **(K, M)** Transwell assay in MCF-7 cells. **(L, N)** Transwell assay in MDA-MB-435S cells. ‘*’ indicates *P*-value < 0.05; ‘**’ indicates *P*-value < 0.01; ‘***’ indicates *P*-value < 0.001. "ns" represents non-significant.

**Figure 13:**
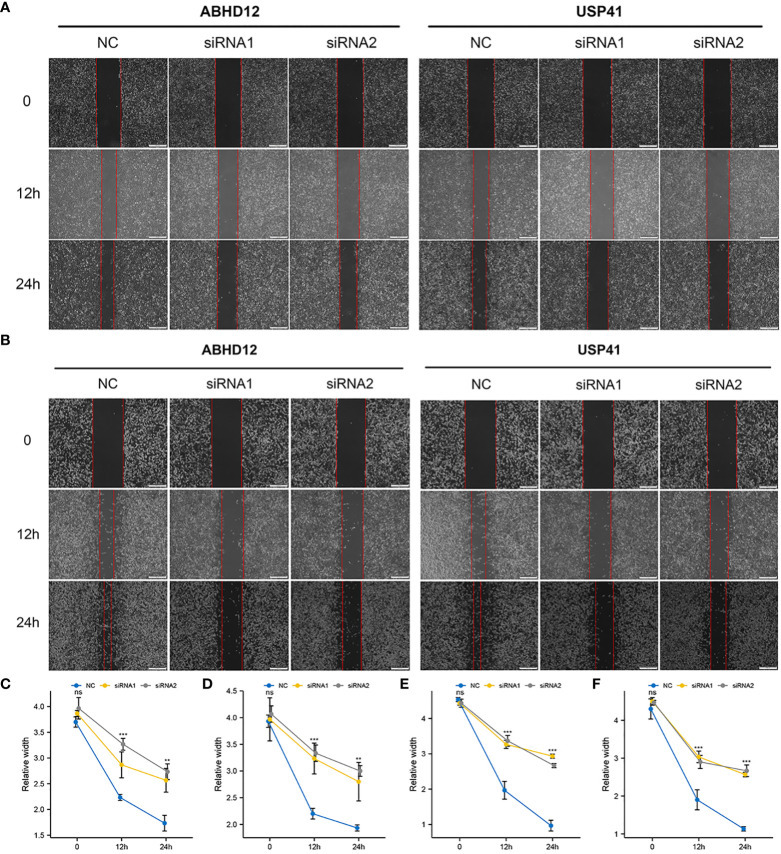
Effects of ABHD12 and USP41 on cell invasion. **(A, C, D)** Wound healing assay in MCF-7 cells. **(B, E, F)** Wound healing assay in MDA-MB-435S cells. ‘**’ indicates *P*-value < 0.01; ‘***’ indicates *P*-value < 0.001. "ns" represents non-significant.

To examine the molecular functions of DRGs in breast cancer, ABHD12 and USP41 were selected for *in vitro* analysis. ABHD12 and USP41 were significantly elevated in both BRCA samples and the high-risk group. ABHD12 and USP41 were knocked down in both MCF-7 cells and MDA-MB-435S cells *via* siRNA transfection (**Figures 12A, B**). Colony formation assay (**Figures 12C–F**) and CCK8 assay (**Figures 12G–J**) consistently verified that the knockdown of ABHD12 and USP41 significantly attenuated the proliferation of both MCF-7 cells and MDA-MB-435S cells. Transwell assay suggested that the knockdown of ABHD12 and USP41 significantly inhibited the migration of both MCF-7 cells and MDA-MB-435S cells (**Figures 12K–N**). Besides, wound healing assay revealed that the knockdown of ABHD12 and USP41 significantly weakened the invasion of both MCF-7 cells and MDA-MB-435S cells (**Figures 13A–F**).

## 4 Discussion

Previously, degradation was merely considered as a destructive mechanism of proteins. Recent studies have revealed that the repertoire of proteases plays an important role in various physiological and pathological activities than ever (19). The identified 625 DRGs account for over 3% of the currently known 19,587 protein-coding genes in human. Therefore, the degradome contains the most abundant class of enzymes that play an essential role in modulating cellular activities (18, 20, 21). Dysregulation of DRGs may contribute to the onset and progression of breast cancer (14–17, 22). But the crucial DRGs remain unknown in breast cancer. Moreover, to the best of our knowledge, no previous study has systematically evaluated the degradome pattern in breast cancer to predict prognosis, assess treatment response and guide risk stratification. Therefore, this study may serve as a primary reference for subsequent studies.

We constructed and validated a 10-gene signature based on DRGs to predict the prognosis of patients with breast cancer. The OS, DSS, DFI and PFI of patients between the low- and high-risk groups were adequately differentiated by the degradome signature. Three DRGs (PRSS2, SPPL2C and RHBDL1) were identified as protective factors and two DRGs (USP41 and ABHD12) were identified as risky factors for BRCA. *In vitro* experiments revealed that USP41 and ABHD12 play an essential role in breast cancer progression, which was consistent with the results of our functional assays (23, 24). Elucidating the detailed regulatory mechanisms of USP41 and ABHD12 may help to further understand their roles in breast cancer. But no previous study has reported the role of PRSS2, SPPL2C and RHBDL1 in breast cancer, thus this study may report the potential role of these genes as independent prognostic predictors in breast cancer for the first time.

Furthermore, the correlation between risk score and clinicopathological parameters was investigated. Patients with T4 stage, ER-negative, PR-negative, HER2-positive, basal subtype (PAM50), Her2 subtype (PAM50), post-menopausal status and those without complete response to chemotherapy harbored high risk score and hence worse prognosis. The risk score was identified as an independent prognostic predictor, together with ER status (negative), N stage (N3) and age. A novel prognostic nomogram integrating these four factors was developed. The C-index, calibration curves and DCA confirmed that the nomogram shows favourable capability to predict survival and clinical decision-making advantages comparing with the traditional pathological stage.

The proportion of DEGs was lower in the low-risk group than in the high-risk group, which led to unsatisfactory results of GO/KEGG functional enrichment analyses and GSEA in the low-risk group. The GO/KEGG biological activities positively upregulated in the high-risk group included small-molecule transport, digestion and peptidase activity, which indicated a more active degradome in the high-risk group. GSEA revealed that the most enriched biological activity in the high-risk group was regulation of TLRs by endogenous ligands. Endogenous ligands from host cell origin that regulate TLRs are also called damage-associated molecular patterns (DAMPs), which can be activated and secreted to respond to tissue damage by enhancing inflammatory responses (25). DAMPs from breast cancer cells and other invasive cancer cells can promote cancer progression and enhance tumor aggressiveness (26, 27), which may explain the worse outcomes of patients in the high-risk group in this study. Furthermore, five cell cycle-promoting biological processes and signaling pathways were enriched in the high-risk group: REG cascade of cyclin EXPR, polo-like kinase activity, G0 and early G1, PLK1 pathway and G1 specific transcription. Polo-like kinase 1 (PLK1) is a pivotal regulator in mitosis. Its overexpression during mitosis activates the transcription factor FOXM1, which subsequently activates genes that are involved in mitosis (28). PLK1 silencing attenuates cell proliferation and growth and induces apoptosis in breast cancer (29, 30). Therefore, the poor prognosis of patients with high risk score in this study may be partially attributed to enhanced proliferation *via* more active mitosis. Additionally, the expression of DRGs was different between the two risk groups, suggesting distinct degradome pattern in the two groups. Altogether, the results of functional enrichment analyses revealed two potential mechanisms underlying breast cancer progression in the high-risk group. On the one hand, DAMPs from cancer cells are activated and secreted to generate inflammation responses and promote tissue repair, thereby enhancing cancer progression. On the other hand, stronger PLK1 signaling enhances mitosis to promote the proliferation of cancer cells. The two mechanisms may provide novel insights into targeted therapy; however, further experimental verification is required to support these findings.

Excessive gene mutation, especially the mutation of tumour suppressor genes, is one of the triggers for tumorigenesis (31). PIK3CA and TP53 are both commonly mutated oncogenes in breast cancer (32). In this study, PIK3CA and TP53 were identified as potential carcinogenesis-driving genes in the low- and high-risk groups, respectively. Kotoula et al. (33) showed that patients with non-lymphocyte-dominant early-stage breast cancer with PIK3CA-only mutations had a favourable DFI, those with TP53-only mutations had a worse DFI and those with PIK3CA-TP53 co-mutations had the worst DFI. They concluded that PIK3CA and TP53 mutations have diverse effects on the prognosis of patients with early stage breast cancer (33). Consistently, in this study, patients with TP53 mutations in the high-risk group had a worse prognosis than those with PIK3CA mutations in the low-risk group. Patients in the high-risk group had higher genome instability and mutation frequency, which may explain the unsatisfactory outcomes observed in this group. More importantly, the risk score was significantly positively correlated with TMB.

Immune infiltrating cells in the TME can influence the prognosis of patients with cancer (34–36). High infiltration levels of T cells usually represent favourable survival probability (37, 38). In breast cancer, CD4 T cells can mitigate CD8 T cell exhaustion, and high infiltration levels of CD4 and CD8 T cells indicate favourable prognosis (39, 40). Consistently, in this study, the infiltration levels of both resting memory CD8 and CD4 T cells were high in the low-risk group with better survival outcomes. Gamma-delta T cells act like a double-edged sword in breast cancer. The Vγ9Vδ2^+^ subtype can exert cytotoxic effects on cancer cells to suppress tumour growth and angiogenesis and induce apoptosis, whereas the γδ1^+^ subtype orchestrates cancer progression (41). In this study, although the infiltration levels of gamma-delta T cells were found to be high in the low-risk group with better prognosis, the subtypes were not identified. Therefore, more precise sequencing data are required for further investigation, such as single-cell sequencing data. NK cells are the main effectors against cancer cells in innate immunity and are correlated with better survival (42). The anti-cancer effects of NK cells activated by ILs have been verified in previous studies (43–45). In this study, the infiltration levels of NK cells were found to be higher in the low-risk group, which verifies the tumour-suppressing role of NK cells. Macrophages are important components of the innate immune system; however, when they infiltrate the TME, called tumour-associated macrophages (TAMs), they are employed by tumour cells to promote cancer progression, resulting in a worse clinical outcome (46). TAMs can restrict tumour-associated antigen presentation and attenuate the activation of cytotoxic T lymphocytes (CTLs) while simultaneously promoting the survival, angiogenesis and metastasis of cancer cells (46). Consistently, in this study, the infiltration levels of macrophages were higher in the high-risk group with a poorer prognosis, which verifies the tumour-promoting role of macrophages. In conclusion, the prognostic role of several tumor infiltrating immune cells in BRCA was verified in this study that immune infiltrating cells in the TME are important for cancer status. Recently, immunotherapy has emerged as the first-line anti-cancer strategy. Identification of the expression patterns of immunotherapeutic targets may help to improve the survival of patients (47, 48). Studies have demonstrated that compared with monotherapy, combination therapy with PD1 and CTLA4 inhibitors results in better survival improvement in several cancers (49–52). In this study, the expression levels of PD1, PDL1 and CTLA4 were higher in the low-risk group, suggesting that co-blockade of these molecules represents a new strategy for immune checkpoint blockade therapy. This finding also indicates that the degradome signature can be used to guide clinical treatment.

The aberrant expression of a single DRG has been reported to associate with the therapeutic effects of chemotherapy (17, 53), endocrinotherapy (54) and radiotherapy (55, 56). In this study, the prognosis of patients undergoing endocrinotherapy or radiotherapy was worse in the high-risk group than in the low-risk group, possibly owing to treatment resistance induced by degradome dysfunction. Subsequently, cyclophosphamide and docetaxel were determined as beneficial chemotherapeutic drug agents for patients in the low-risk group, whereas 5-fluorouracil may be more proper for patients in the high-risk group. Additionally, evaluation of the expression pattern of potential molecular targets revealed that targeting CDKs/PARPs may represent a better therapeutic strategy for patients in the high-risk group, which is consistent with the results of functional enrichment analysis. In the meanwhile, targeting the PI3K/AKT/mTOR signaling pathway may serve as a better therapeutic strategy for patients in the low-risk group, which is consistent with the results of mutation analysis. These findings also indicate the utility of the degradome signature in developing individualized treatment strategies in clinical settings.

However, this study has certain limitations. First, specimens from real-world clinical patients are required for verifying the expression of the 10 DRGs. Second, prospective, multi-centre studies with a large BRCA cohort should be conducted to verify the reliability of the degradome signature and corresponding results. Third, more experimental studies are required to elucidate the regulatory mechanisms and functions of the 10 DRGs.

## 5 Conclusion

A 10-gene signature based on DRGs was constructed and validated to predict the prognosis of breast cancer. A nomogram integrating clinicopathological parameters and risk score was further developed for predicting OS. The high-risk group had a higher degree of clinicopathological events, as well as higher mutation frequency. TLR regulation and several cell cycle-promoting activities were significantly upregulated in the high-risk group. The risk score was significantly correlated with the infiltration of several immune cells and TMB. The expression of various immune checkpoints, including PD1, PDL1 and CTLA4, was significantly higher in the low-risk group. Additionally, the prognosis of patients undergoing different therapies was distinguished by the degradome signature between the two risk groups possibly owing to treatment resistance. Therefore, the degradome signature may be utilized for prognostic prediction, risk stratification and clinical decision making in breast cancer.

## Data availability statement

The datasets presented in this study can be found in online repositories. The names of the repository/repositories and accession number(s) can be found in the article/**Supplementary Material**.

## Ethics statement

Consent from all participants was obtained through The Cancer Genome Atlas (TCGA) database, the Molecular Taxonomy of Breast Cancer International Consortium (METABRIC) and the Gene Expression Omnibus (GEO) database.

## Author contributions

YL processed the bioinformatic analyses, and YY performed the functional assays. YL, YY, YC, CZ and YS drafted and edited the manuscript. CW gave final approval of the revised manuscript. JO outlined the study and edited the manuscript. All authors contributed to the article and approved the submitted version.

## References

[1] Sung H Ferlay J Siegel RL Laversanne M Soerjomataram I Jemal A. Global cancer statistics 2020: GLOBOCAN estimates of incidence and mortality worldwide for 36 cancers in 185 countries. CA Cancer J Clin (2021) 71(3):209–49. doi: 10.3322/caac.21660 33538338

[2] Xie J Luo X Deng X Tang Y Tian W Cheng H. Advances in artificial intelligence to predict cancer immunotherapy efficacy. Front Immunol (2023) 13:1076883. doi: 10.3389/fimmu.2022.1076883 36685496 PMC9845588

[3] Harbeck N Penault-Llorca F Cortes J Gnant M Houssami N Poortmans P. Breast cancer. Nat Rev Dis Primers (2019) 5(1):66. doi: 10.1038/s41572-019-0111-2 31548545

[4] Li W Wang H Dong S Tang ZR Chen L Cai X. Establishment and validation of a nomogram and web calculator for the risk of new vertebral compression fractures and cement leakage after percutaneous vertebroplasty in patients with osteoporotic vertebral compression fractures. Eur Spine J (2022) 31(5):1108–21. doi: 10.1007/s00586-021-07064-z 34822018

[5] Li W Dong S Wang B Wang H Xu C Zhang K. The construction and development of a clinical prediction model to assess lymph node metastases in osteosarcoma. Front Public Health (2022) 9:813625. doi: 10.3389/fpubh.2021.813625 35071175 PMC8770939

[6] Dong S Li W Tang ZR Wang H Pei H Yuan B. Development and validation of a novel predictive model and web calculator for evaluating transfusion risk after spinal fusion for spinal tuberculosis: a retrospective cohort study. BMC Musculoskelet Disord (2021) 22(1):825. doi: 10.1186/s12891-021-04715-6 34563170 PMC8466716

[7] Wang H Tang ZR Li W Fan T Zhao J Kang M. Prediction of the risk of C5 palsy after posterior laminectomy and fusion with cervical myelopathy using a support vector machine: an analysis of 184 consecutive patients. J Orthop Surg Res (2021) 16(1):332. doi: 10.1186/s13018-021-02476-5 34020677 PMC8139086

[8] Dong S Yang H Tang ZR Ke Y Wang H Li W. Development and validation of a predictive model to evaluate the risk of bone metastasis in kidney cancer. Front Oncol (2021) 11:731905. doi: 10.3389/fonc.2021.731905 34900681 PMC8656153

[9] Puente XS Sánchez LM Overall CM López-Otín C. Human and mouse proteases: a comparative genomic approach. Nat Rev Genet (2003) 4(7):544–58. doi: 10.1038/nrg1111 12838346

[10] Müller SA Scilabra SD Lichtenthaler SF. Proteomic substrate identification for membrane proteases in the brain. Front Mol Neurosci (2016) 9:96. doi: 10.3389/fnmol.2016.00096 27790089 PMC5062031

[11] Wünnemann F Ta-Shma A Preuss C Leclerc S van Vliet PP Oneglia A. Loss of ADAMTS19 causes progressive non-syndromic heart valve disease. Nat Genet (2020) 52(1):40–7. doi: 10.1038/s41588-019-0536-2 PMC7197892 31844321

[12] Krane SM. Elucidation of the potential roles of matrix metalloproteinases in skeletal biology. Arthritis Res Ther (2003) 5(1):2–4. doi: 10.1186/ar600 12716440 PMC154421

[13] de Bruyn M Vandooren J Ugarte-Berzal E Arijs I Vermeire S Opdenakker G. The molecular biology of matrix metalloproteinases and tissue inhibitors of metalloproteinases in inflammatory bowel diseases. Crit Rev Biochem Mol Biol (2016) 51(5):295–358. doi: 10.1080/10409238.2016.1199535 27362691

[14] Geng N Li Y Zhang W Wang F Wang X Jin Z. A PAK5-DNPEP-USP4 axis dictates breast cancer growth and metastasis. Int J Cancer (2020) 146(4):1139–51. doi: 10.1002/ijc.32523 31219614

[15] Murray AS Hyland TE Sala-Hamrick KE Mackinder JR Martin CE Tanabe LM. The cell-surface anchored serine protease TMPRSS13 promotes breast cancer progression and resistance to chemotherapy. Oncogene (2020) 39(41):6421–36. doi: 10.1038/s41388-020-01436-3 PMC8143875 32868877

[16] Dong Y Van Tine BA Oyama T Wang PI Cheng EH Hsieh JJ. Taspase1 cleaves MLL1 to activate cyclin e for HER2/neu breast tumorigenesis. Cell Res (2014) 24(11):1354–66. doi: 10.1038/cr.2014.129 PMC4220155 25267403

[17] Radisky ES Radisky DC. Matrix metalloproteinases as breast cancer drivers and therapeutic targets. Front Biosci (Landmark Ed) (2015) 20(7):1144–63. doi: 10.2741/4364 PMC4516284 25961550

[18] Pérez-Silva JG Español Y Velasco G Quesada V. The degradome database: expanding roles of mammalian proteases in life and disease. Nucleic Acids Res (2016) 44(D1):D351–5. doi: 10.1093/nar/gkv1201 PMC4702854 26553809

[19] López-Otín C Overall CM. Protease degradomics: a new challenge for proteomics. Nat Rev Mol Cell Biol (2002) 3(7):509–19. doi: 10.1038/nrm858 12094217

[20] Ezkurdia I Juan D Rodriguez JM Frankish A Diekhans M Harrow J. Multiple evidence strands suggest that there may be as few as 19,000 human protein-coding genes. Hum Mol Genet (2014) 23(22):5866–78. doi: 10.1093/hmg/ddu309 PMC4204768 24939910

[21] Omenn GS Lane L Lundberg EK Beavis RC Overall CM Deutsch EW. Metrics for the human proteome project 2016: Progress on identifying and characterizing the human proteome, including post-translational modifications. J Proteome Res (2016) 15(11):3951–60. doi: 10.1021/acs.jproteome.6b00511 PMC5129622 27487407

[22] Osuala KO Ji K Mattingly RR Sloane BF. Breast cancer: Proteolysis and migration. Adv Exp Med Biol (2019) 1152:401–11. doi: 10.1007/978-3-030-20301-6_21 31456196

[23] Huang M Xiao J Yan C Wang T Ling R. USP41 promotes breast cancer *via* regulating RACK1. Ann Transl Med (2021) 9(20):1566. doi: 10.21037/atm-21-4921 34790772 PMC8576695

[24] Jun S SW K JY L Park SJ. ABHD12 knockdown suppresses breast cancer cell proliferation, migration and invasion. Anticancer Res (2020) 40(5):2601–11. doi: 10.21873/anticanres.14231 32366405

[25] Erridge C. Endogenous ligands of TLR2 and TLR4: agonists or assistants? J Leukoc Biol (2010) 87(6):989–99. doi: 10.1189/jlb.1209775 20179153

[26] Eteshola EOU Landa K Rempel RE Naqvi IA Hwang ES Nair SK. Breast cancer-derived DAMPs enhance cell invasion and metastasis, while nucleic acid scavengers mitigate these effects. Mol Ther Nucleic Acids (2021) 26:1–10. doi: 10.1016/j.omtn.2021.06.016 34513289 PMC8408553

[27] Garg AD Agostinis P. Cell death and immunity in cancer: From danger signals to mimicry of pathogen defense responses. Immunol Rev (2017) 280(1):126–48. doi: 10.1111/imr.12574 29027218

[28] Reactome. Polo-like kinase mediated events (2022). Available at: http://www.reactome.org/content/detail/R-HSA-156711 (Accessed September 30, 2022).

[29] Yao YD Sun TM Huang SY Dou S Lin L Chen JN. Targeted delivery of PLK1-siRNA by ScFv suppresses Her2+ breast cancer growth and metastasis. Sci Transl Med (2012) 4(130):130ra48. doi: 10.1126/scitranslmed.3003601 22517885

[30] Pan Z Chen Y Liu J Jiang Q Yang S Guo L. Design, synthesis, and biological evaluation of polo-like kinase 1/eukaryotic elongation factor 2 kinase (PLK1/EEF2K) dual inhibitors for regulating breast cancer cells apoptosis and autophagy. Eur J Med Chem (2018) 144:517–28. doi: 10.1016/j.ejmech.2017.12.046 29288948

[31] Vogelstein B Papadopoulos N Velculescu VE Zhou S Diaz LA Jr Kinzler KW. Cancer genome landscapes. Science (2013) 339(6127):1546–58. doi: 10.1126/science.1235122 PMC3749880 23539594

[32] Cancer Genome Atlas Network. Comprehensive molecular portraits of human breast tumours. Nature (2012) 490(7418):61–70. doi: 10.1038/nature11412 23000897 PMC3465532

[33] Kotoula V Karavasilis V Zagouri F Kouvatseas G Giannoulatou E Gogas H. Effects of TP53 and PIK3CA mutations in early breast cancer: a matter of co-mutation and tumor-infiltrating lymphocytes. Breast Cancer Res Treat (2016) 158(2):307–21. doi: 10.1007/s10549-016-3883-z 27369359

[34] Ino Y Yamazaki-Itoh R Shimada K Iwasaki M Kosuge T Kanai Y. Immune cell infiltration as an indicator of the immune microenvironment of pancreatic cancer. Br J Cancer (2013) 108(4):914–23. doi: 10.1038/bjc.2013.32 PMC3590668 23385730

[35] Zhang L Conejo-Garcia JR Katsaros D Gimotty PA Massobrio M Regnani G. Intratumoral T cells, recurrence, and survival in epithelial ovarian cancer. N Engl J Med (2003) 348(3):203–13. doi: 10.1056/NEJMoa020177 12529460

[36] Xie J Zheng S Zou Y Tang Y Tian W Wong CW. Turning up a new pattern: Identification of cancer-associated fibroblast-related clusters in TNBC. Front Immunol (2022) 13:1022147. doi: 10.3389/fimmu.2022.1022147 36275659 PMC9583405

[37] Pagès F Berger A Camus M Sanchez-Cabo F Costes A Molidor R. Effector memory T cells, early metastasis, and survival in colorectal cancer. N Engl J Med (2005) 353(25):2654–66. doi: 10.1056/NEJMoa051424 16371631

[38] Sato E Olson SH Ahn J Bundy B Nishikawa H Qian F. Intraepithelial CD8+ tumor-infiltrating lymphocytes and a high CD8+/regulatory T cell ratio are associated with favorable prognosis in ovarian cancer. Proc Natl Acad Sci U.S.A. (2005) 102(51):18538–43. doi: 10.1073/pnas.0509182102 PMC1311741 16344461

[39] Kmieciak M Worschech A Nikizad H Gowda M Habibi M Depcrynski A. CD4+ T cells inhibit the neu-specific CD8+ T-cell exhaustion during the priming phase of immune responses against breast cancer. Breast Cancer Res Treat (2011) 126(2):385–94. doi: 10.1007/s10549-010-0942-8 PMC3086038 20480224

[40] Matsumoto H Thike AA Li H Yeong J Koo SL Dent RA. Increased CD4 and CD8-positive T cell infiltrate signifies good prognosis in a subset of triple-negative breast cancer. Breast Cancer Res Treat (2016) 156(2):237–47. doi: 10.1007/s10549-016-3743-x 26960711

[41] Morrow ES Roseweir A Edwards J. The role of gamma delta T lymphocytes in breast cancer: a review. Transl Res (2019) 203:88–96. doi: 10.1016/j.trsl.2018.08.005 30194922

[42] Wu SY Fu T Jiang YZ Shao ZM. Natural killer cells in cancer biology and therapy. Mol Cancer (2020) 19(1):120. doi: 10.1186/s12943-020-01238-x 32762681 PMC7409673

[43] Widowati W Jasaputra DK Sumitro SB Widodo MA Mozef T Rizal R. Effect of interleukins (IL-2, IL-15, IL-18) on receptors activation and cytotoxic activity of natural killer cells in breast cancer cell. Afr Health Sci (2020) 20(2):822–32. doi: 10.4314/ahs.v20i2.36 PMC7609126 33163049

[44] Juliá EP Mordoh J Levy EM. Cetuximab and IL-15 promote NK and dendritic cell activation *In vitro* in triple negative breast cancer. Cells (2020) 9(7):1573. doi: 10.3390/cells9071573 32605193 PMC7408037

[45] Asgari A Sharifzadeh S Ghaderi A Hosseini A Ramezani A. *In vitro* cytotoxic effect of trastuzumab in combination with pertuzumab in breast cancer cells is improved by interleukin-2 activated NK cells. Mol Biol Rep (2019) 46(6):6205–13. doi: 10.1007/s11033-019-05059-0 31493284

[46] Mehta AK Kadel S Townsend MG Oliwa M Guerriero JL. Macrophage biology and mechanisms of immune suppression in breast cancer. Front Immunol (2021) 12:643771. doi: 10.3389/fimmu.2021.643771 33968034 PMC8102870

[47] Cao Z Zhang S. An integrative and comparative study of pan-cancer transcriptomes reveals distinct cancer common and specific signatures. Sci Rep (2016) 6:33398. doi: 10.1038/srep33398 27633916 PMC5025752

[48] Xie J Zou Y Gao T Xie L Tan D Xie X. Therapeutic landscape of human epidermal growth factor receptor 2-positive breast cancer. Cancer Control (2022) 29:10732748221099230. doi: 10.1177/10732748221099230 35499382 PMC9067050

[49] Hammers HJ Plimack ER Infante JR Rini BI McDermott DF Lewis LD. Safety and efficacy of nivolumab in combination with ipilimumab in metastatic renal cell carcinoma: The CheckMate 016 study. J Clin Oncol (2017) 35(34):3851–8. doi: 10.1200/JCO.2016.72.1985 PMC7587408 28678668

[50] Hellmann MD Rizvi NA Goldman JW Gettinger SN Borghaei H Brahmer JR. Nivolumab plus ipilimumab as first-line treatment for advanced non-small-cell lung cancer (CheckMate 012): results of an open-label, phase 1, multicohort study. Lancet Oncol (2017) 18(1):31–41. doi: 10.1016/S1470-2045(16)30624-6 27932067 PMC5476941

[51] Larkin J Chiarion-Sileni V Gonzalez R Grob JJ Cowey CL Lao CD. Combined nivolumab and ipilimumab or monotherapy in untreated melanoma. N Engl J Med (2015) 373(1):23–34. doi: 10.1056/NEJMoa1504030 26027431 PMC5698905

[52] Wolchok JD Kluger H Callahan MK Postow MA Rizvi NA Lesokhin AM. Nivolumab plus ipilimumab in advanced melanoma. N Engl J Med (2013) 369(2):122–33. doi: 10.1056/NEJMoa1302369 PMC5698004 23724867

[53] Ozdemir K Zengin I Guney Eskiler G Kocer HB Ozkan AD Demiray T. The predictive role of MMP-2, MMP-9, TIMP-1 and TIMP-2 serum levels in the complete response of the tumor to chemotherapy in breast cancer patients. J Invest Surg (2022) 35(7):1544–50. doi: 10.1080/08941939.2022.2080308 35636767

[54] Li Q Zan L. Knockdown of ATG4A inhibits breast cancer progression and promotes tamoxifen chemosensitivity by suppressing autophagy. Mol Med Rep (2022) 25(3):101. doi: 10.3892/mmr.2022.12617 35088889 PMC8822883

[55] Ager EI Kozin SV Kirkpatrick ND Seano G Kodack DP Askoxylakis V. Blockade of MMP14 activity in murine breast carcinomas: implications for macrophages, vessels, and radiotherapy. J Natl Cancer Inst (2015) 107(4):djv017. doi: 10.1093/jnci/djv017 25710962 PMC4402365

[56] Langlands FE Dodwell D Hanby AM Horgan K Millican-Slater RA Speirs V. PSMD9 expression predicts radiotherapy response in breast cancer. Mol Cancer (2014) 13:73. doi: 10.1186/1476-4598-13-73 24673853 PMC4230020

