# Supplementary material for: A degradome-based prognostic signature that correlates with immune infiltration and tumor mutation burden in breast cancer
Source: Front Immunol. 2023 Mar 13;14:1140993. doi: 10.3389/fimmu.2023.1140993 (PMC10040797; doi:10.3389/fimmu.2023.1140993)

***Supplementary Materials***

ABHD12 siRNA1:

sense (5’-3’): GCCACCCUAUCAUUCUGUATT

antisense (5’-3’): UACAGAAUGAUAGGGUGGCTT

ABHD12 siRNA2:

sense (5’-3’): GGAAUCUCCAUUCACUAAUTT

antisense (5’-3’): AUUAGUGAAUGGAGAUUCCTT

USP41 siRNA1:

sense (5’-3’): GCUGGGAGCAUGAGUUUAUTT

antisense (5’-3’): AUAAACUCAUGCUCCCAGCTT

USP41 siRNA2:

sense (5’-3’): GGUGCAGAAUUGAUAUUAUTT

antisense (5’-3’): AUAAUAUCAAUUCUGCACCTT

**Supplementary Figure S1.** Consensus clustering of TCGA cohort based on 22 degradome-related genes (DRGs). **(A-C)** Consensus clustering. **(D)** Expression pattern of the 22 DRGs between BRCA subtype 1 and BRCA subtype 2. **(E)** Survival difference between BRCA subtype 1 and BRCA subtype 2.

**
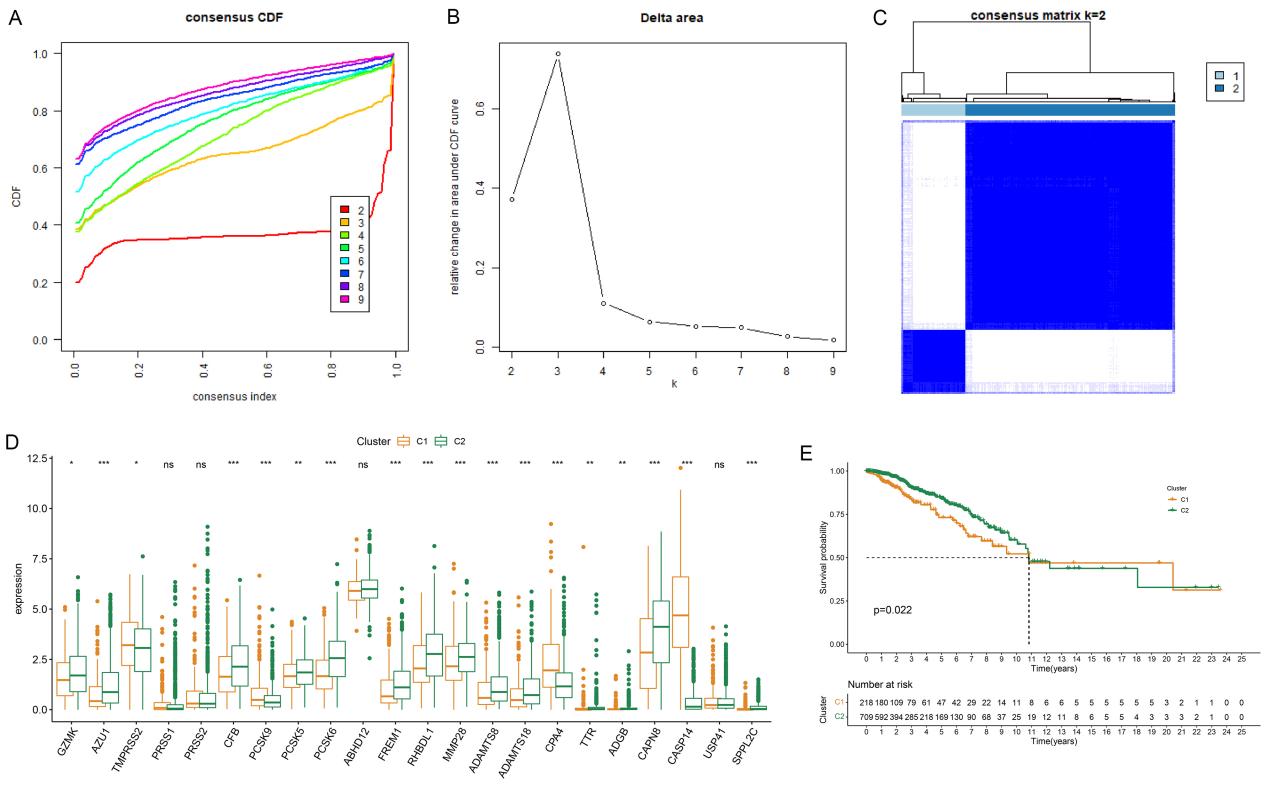
**

**Supplementary Figure S2.** Principal component analysis. **(A)** Principal component analysis between low-risk group and high-risk group in TCGA cohort. **(B)** Principal component analysis between low-risk group and high-risk group in GSE96058.


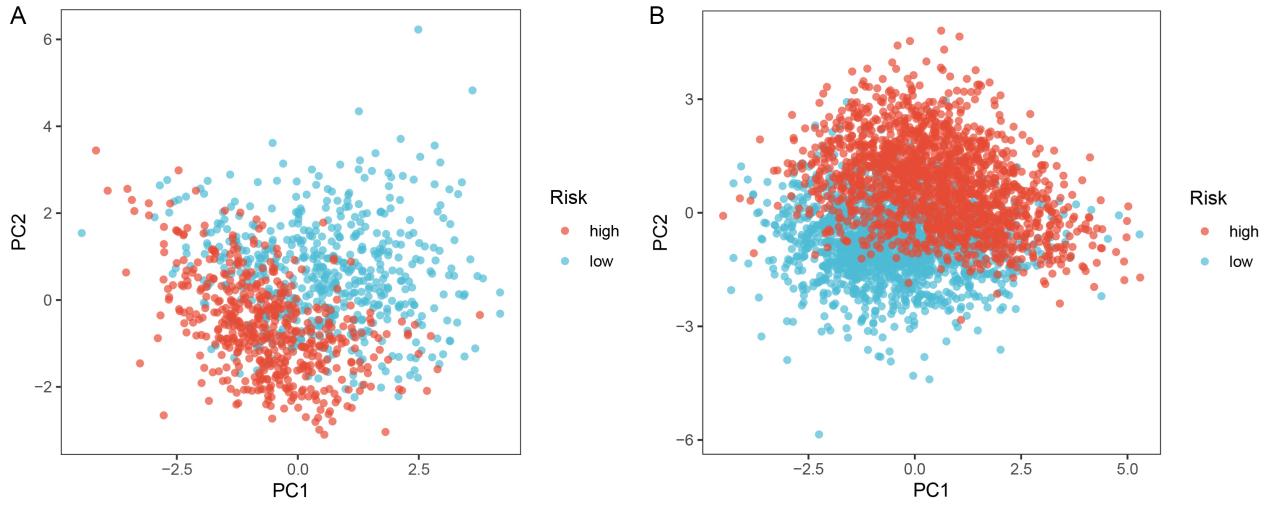


**Supplementary Figure S3.** ROC curves showing the predictive capability of risk score and other clinicopathological characteristics. **(A)** The predictive capability of risk score in TCGA cohort. **(B)** The predictive capability of risk score in GSE96058.

**
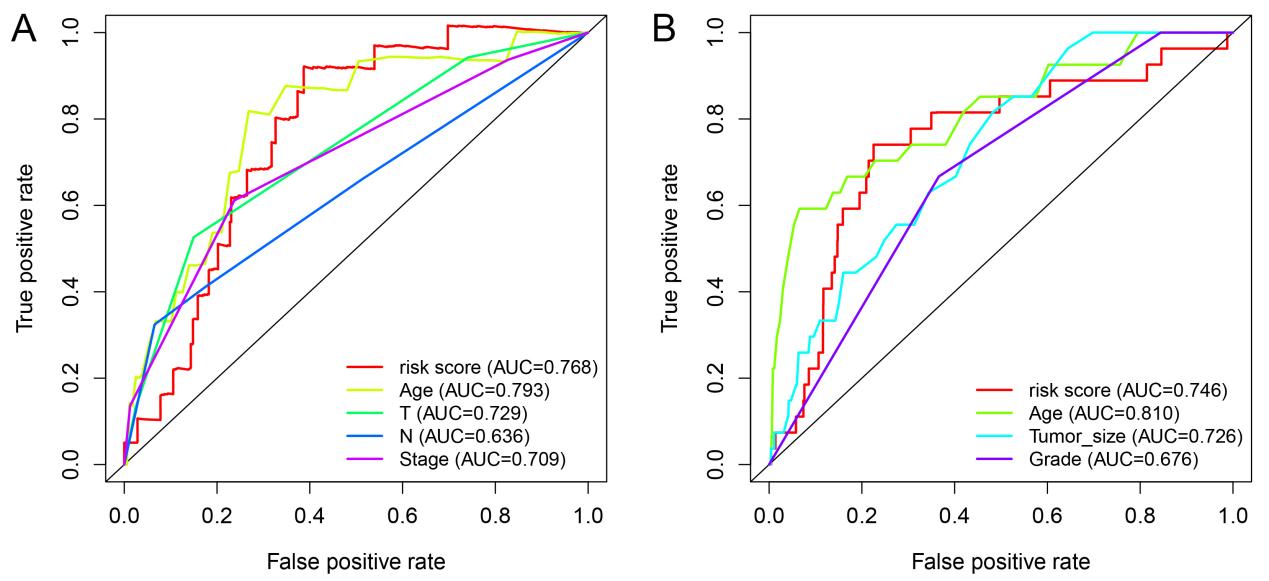
**

**Supplementary Figure S4.** Subgroup analysis of the degradome signature. **(A)** Age: ≤ 65 & > 65. **(B)** Anatomic location of primary tumor: Right & Left. **(C)** T stage: T_1-2_ & T_3-4_. **(D)** Lymph node status: Positive & Negative. **(E)** Person neoplasm cancer status: Tumor free & With tumor. **(F)** Clinical stage: Early breast cancer & Advanced breast cancer. **(G)** Histology: Infiltrating ductal carcinoma & Infiltrating lobular carcinoma. **(H)** PAM50: Luminal & Basal (TNBC). **(I)** Menopause: Post & Pre.

**
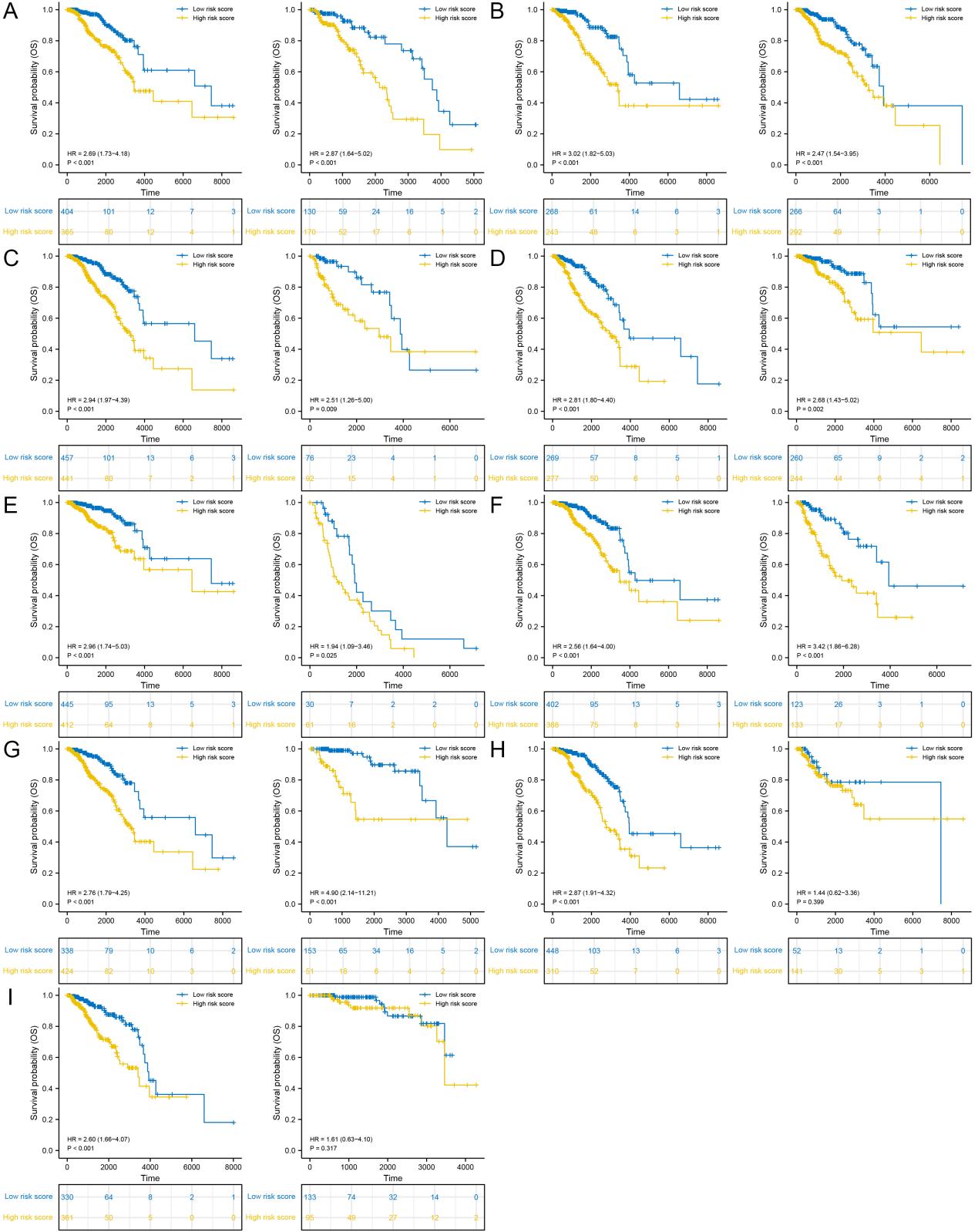
**

**Supplementary Figure S5.** Univariate and multivariate Cox regression analyses of the four validation cohorts. **(A-B)** TCGA ER-positive cohort. **(C-D)** TCGA HER2-positive cohort. **(E-F)** TCGA Pathological stage III. **(G-H)** GSE96058.


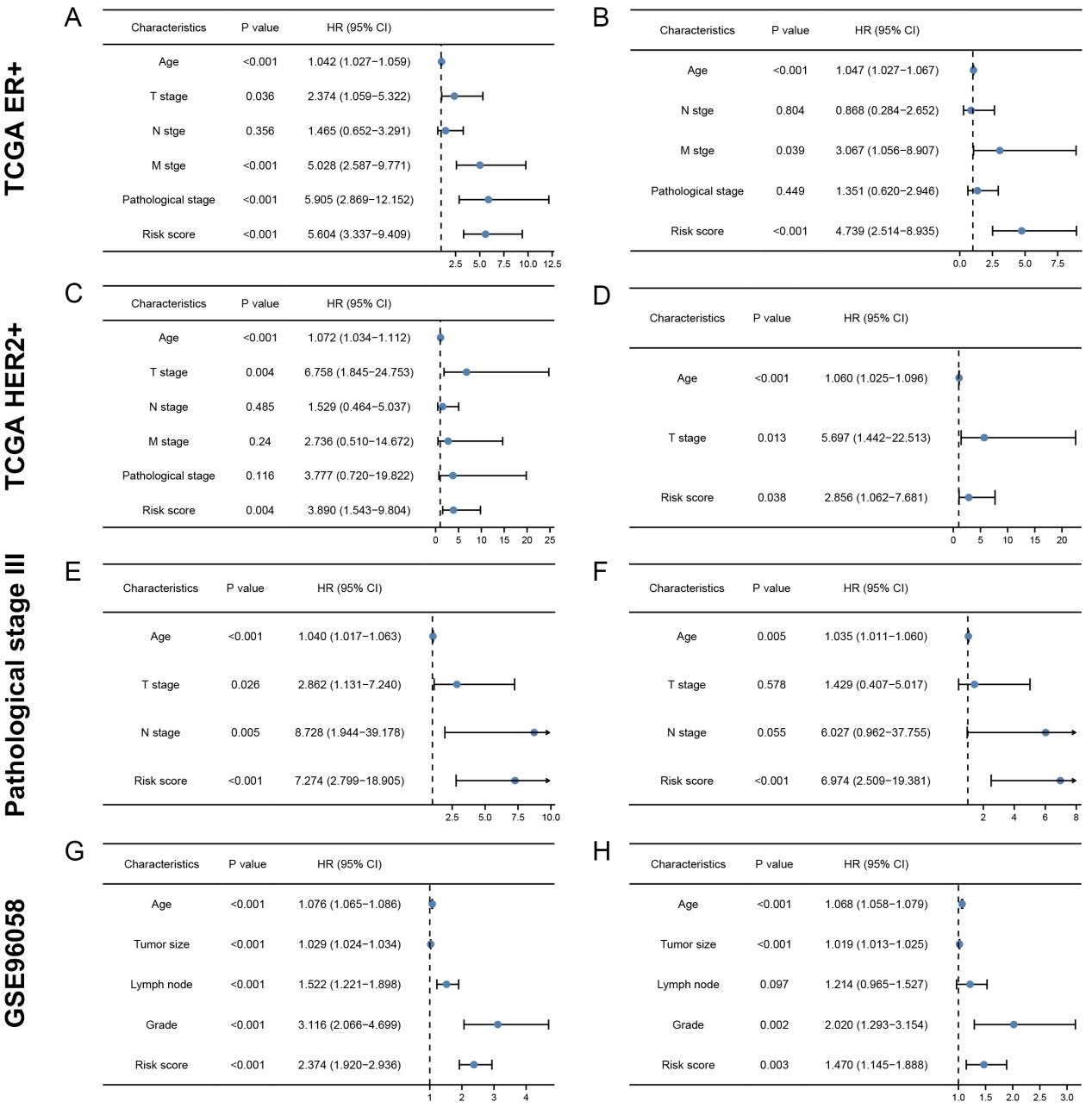


**Supplementary Figure S6.** Drug sensitivity analysis with IC50. (A) Docetaxel. (B) Epirubicin. (C) Afuresertib. (D) Buparlisib. (E) Ipatasertib. (F) Dactolisib. (G) Ibrutinib. (H) Lapatinib. (I) Sapitinib.


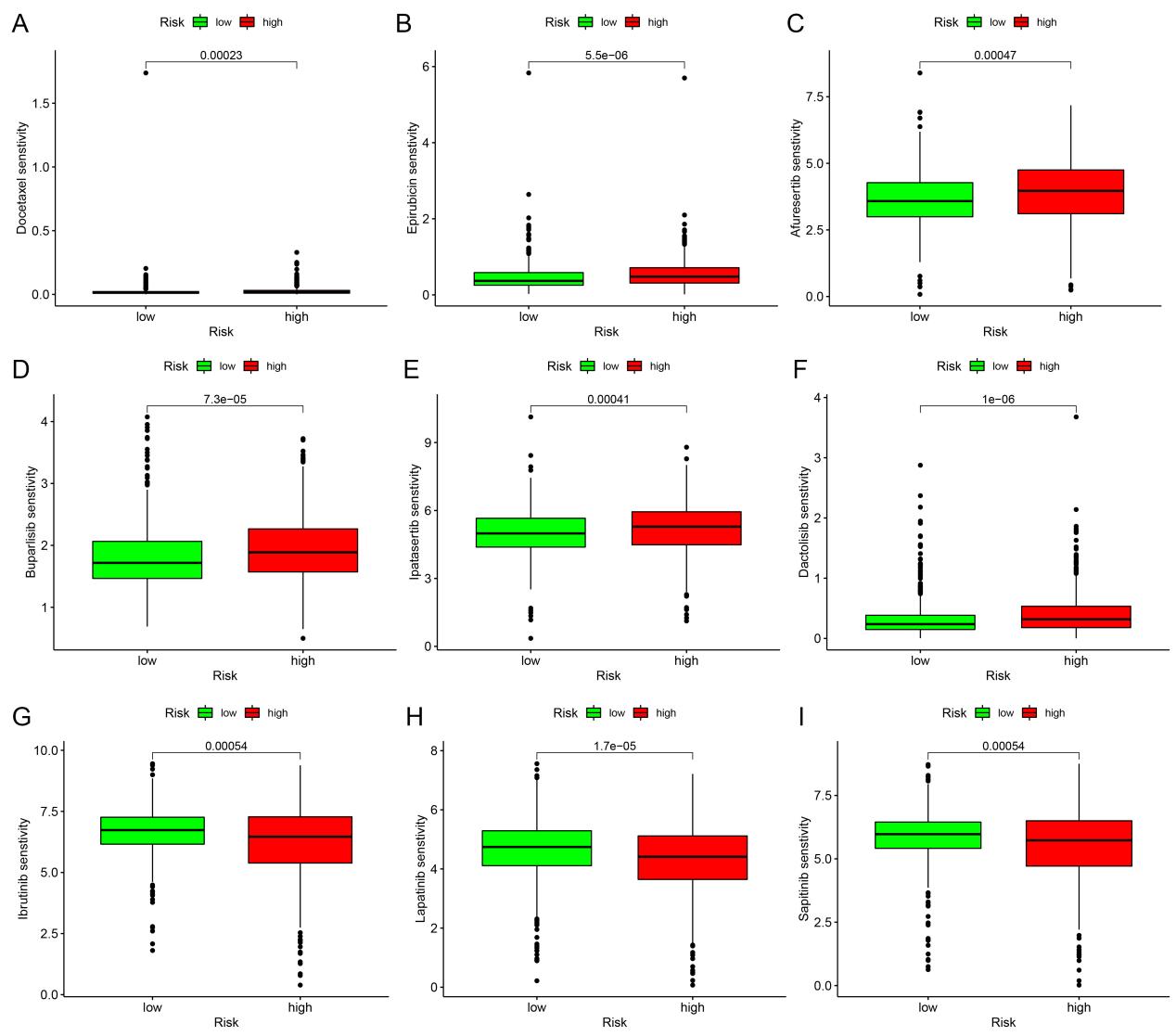

Supplement: Supplementary file 1 [file DataSheet_1.docx]
