# Supplementary material for: A degradome-based prognostic signature that correlates with immune infiltration and tumor mutation burden in breast cancer
Source: Front Immunol. 2023 Mar 13;14:1140993. doi: 10.3389/fimmu.2023.1140993 (PMC10040797; doi:10.3389/fimmu.2023.1140993)
Supplement: Supplementary file 2 [file DataSheet_2.docx]

***Supplementary Materials***

**Supplementary Table S1.** Differences of clinicopathological characteristics between low-risk group and high-risk group.

| Clinicopathological characteristics | Low-risk group (N) | High-risk group (N) | χ^2^ value | *P*-value |
| --- | --- | --- | --- | --- |
| **Age** | 534 | 535 |  |  |
| ≤ 40 | 53 (9.93%) | 46 (8.60%) | 0.56 | *P* > 0.05 |
| > 40 | 481 (90.07%) | 489 (91.40%) | 0.56 | *P* > 0.05 |
| **Race** | 501 | 489 |  |  |
| White | 399 (79.64%) | 353 (72.19%) | 7.53 | ***P* < 0.01** |
| Black | 77 (15.37%) | 102 (20.86%) | 5.03 | ***P* < 0.025** |
| Asian | 25 (4.99%) | 33 (6.75%) | 1.39 | *P* > 0.05 |
| American Indian | 0 (0%) | 1 (0.20%) | 0.00015 | *P* > 0.05 |
| **Pathological T stage** | 533 | 533 |  |  |
| T1 | 161 (30.21%) | 115 (21.58%) | 10.35 | ***P* < 0.005** |
| T2 | 296 (55.53%) | 326 (61.16%) | 3.47 | *P* > 0.05 |
| T3 | 72 (13.51%) | 63 (11.82%) | 0.69 | *P* > 0.05 |
| T4 | 4 (0.75%) | 29 (5.44%) | 19.54 | ***P* < 0.005** |
| **Pathological N stage** | 529 | 521 |  |  |
| N0 | 260 (49.15%) | 244 (46.83%) | 0.56 | *P* > 0.05 |
| N1 | 179 (33.84%) | 176 (33.78%) | 0.00037 | *P* > 0.05 |
| N2 | 50 (9.45%) | 66 (12.67%) | 2.76 | *P* > 0.05 |
| N3 | 40 (7.56%) | 35 (6.71%) | 0.28 | *P* > 0.05 |
| **Pathological M stage** | 451 | 458 |  |  |
| M0 | 445 (98.67%) | 444 (96.94%) | 3.15 | *P* > 0.05 |
| M1 | 6 (1.33%) | 14 (3.06%) | 3.15 | *P* > 0.05 |
| **Pathological stage** | 525 | 521 |  |  |
| Stage I | 109 (20.76%) | 71 (13.63%) | 9.34 | ***P* < 0.005** |
| Stage II | 293 (55.81%) | 317 (60.84%) | 2.73 | *P* > 0.05 |
| Stage III | 118 (22.48%) | 120 (23.03%) | 0.05 | *P* > 0.05 |
| Stage IV | 5 (0.95%) | 13 (2.50%) | 3.68 | *P* > 0.05 |
| **ER status** | 512 | 511 |  |  |
| Positive | 446 (87.12%) | 335 (65.56%) | 65.78 | ***P* < 0.005** |
| Indeterminate | 0 (0%) | 2 (0.39%) | 0.50 | *P* > 0.05 |
| Negative | 66 (12.89%) | 174 (34.05%) | 9.76 | ***P* < 0.005** |
| **PR status** | 511 | 511 |  |  |
| Positive | 407 (79.65%) | 273 (53.42%) | 78.91 | ***P* < 0.005** |
| Indeterminate | 4 (0.78%) | 0 (0%) | 0.00074 | *P* > 0.05 |
| Negative | 100 (19.57%) | 238 (46.57%) | 84.19 | ***P* < 0.005** |
| **HER2 status** | 441 | 455 |  |  |
| Positive | 48 (10.88%) | 105 (23.08%) | 23.51 | ***P* < 0.005** |
| Indeterminate | 6 (1.36%) | 6 (1.32%) | 0.003 | *P* > 0.05 |
| Equivocal | 100 (22.68%) | 79 (17.36%) | 13.03 | ***P* < 0.005** |
| Negative | 287 (65.08%) | 265 (58.24%) | 4.43 | ***P* < 0.05** |
| **Menopause** | 497 | 496 |  |  |
| Pre | 133 (26.76%) | 95 (19.15%) | 8.12 | ***P* < 0.005** |
| Post | 330 (66.40%) | 361 (72.78%) | 4.78 | ***P* < 0.05** |
| Indeterminate | 16 (3.22%) | 18 (3.63%) | 0.13 | *P* > 0.05 |
| Peri | 18 (3.62%) | 22 (4.44%) | 0.43 | *P* > 0.05 |
| **PAM50** | 534 | 535 |  |  |
| LumA | 383 (71.72%) | 175 (32.71%) | 376.08 | ***P* < 0.005** |
| LumB | 65 (12.17%) | 135 (25.23%) | 29.98 | ***P* < 0.005** |
| Basal | 52 (9.74%) | 141 (26.36%) | 49.98 | ***P* < 0.005** |
| Normal | 24 (4.49%) | 15 (2.80%) | 2.17 | *P* > 0.05 |
| Her2 | 10 (1.87%) | 69 (12.90%) | 47.46 | ***P* < 0.005** |
| **Chemotherapy** | 252 | 239 |  |  |
| Complete response | 94 (37.30%) | 59 (24.69%) | 9.10 | ***P* < 0.005** |
| Non-complete response | 158 (62.70%) | 180 (75.31%) | 9.10 | ***P* < 0.005** |
| **Endocrinotherapy** | 154 | 108 |  |  |
| Complete response | 12 (7.79%) | 4 (3.70%) | 1.85 | *P* > 0.05 |
| Non-complete response | 142 (92.21%) | 104 (96.30%) | 1.85 | *P* > 0.05 |
| **Radiotherapy** | 44 | 28 |  |  |
| Complete response | 27 (61.36%) | 17 (60.71%) | 0.003 | *P* > 0.05 |
| Non-complete response | 17 (38.64%) | 11 (39.29%) | 0.003 | *P* > 0.05 |

**Supplementary Table S2.** Univariate and multivatiate Cox regression analyses of clinicopathological characteristics and risk score.

| Clinicopathological characteristics | Total (N) | Univariate analysis | | Multivariate analysis | |
| --- | --- | --- | --- | --- | --- |
|  |  | HR (95% CI) | *P*-value | HR (95% CI) | *P*-value |
| **Age** | 1069 | 1.031 (1.019-1.044) | < 0.001 | 1.042 (1.018-1.066) | **< 0.001** |
| **Race** | 990 |  | 0.865 |  |  |
| White | 752 | Reference |  |  |  |
| Black | 179 | 1.151 (0.765-1.731) | 0.501 |  |  |
| Asian | 58 | 0.754 (0.239-2.383) | 0.631 |  |  |
| American Indian | 1 | 0.000 (0.000-Inf) | 0.994 |  |  |
| **HER2 status** | 896 |  | 0.116 |  |  |
| Negative | 552 | Reference |  |  |  |
| Positive | 153 | 1.592 (0.973-2.605) | 0.064 |  |  |
| Indeterminate | 12 | 0.000 (0.000-Inf) | 0.994 |  |  |
| Equivocal | 179 | 0.736 (0.423-1.280) | 0.277 |  |  |
| **ER status** | 1018 |  | < 0.001 |  |  |
| Positive | 777 | Reference |  |  |  |
| Negative | 239 | 1.402 (0.976-2.016) | 0.068 | 2.184 (1.056-4.515) | **0.035** |
| Indeterminate | 2 | 18.328 (4.463-75.269) | < 0.001 |  |  |
| **PR status** | 1022 |  | 0.191 |  |  |
| Positive | 680 | Reference |  |  |  |
| Negative | 338 | 1.365 (0.976-1.910) | 0.069 |  |  |
| Indeterminate | 4 | 1.128 (0.156-8.182) | 0.905 |  |  |
| **Pathological stage** | 1046 |  | < 0.001 |  |  |
| Stage II | 610 | Reference |  |  |  |
| Stage I | 180 | 0.589 (0.342-1.015) | 0.057 | 0.422 (0.152-1.175) | 0.099 |
| Stage III | 238 | 1.745 (1.190-2.560) | 0.004 | 1.837 (0.678-4.976) | 0.232 |
| Stage IV | 18 | 6.840 (3.772-12.402) | < 0.001 | 3.139 (0.816-12.067) | 0.096 |
| **Pathological M stage** | 909 |  | < 0.001 |  |  |
| M0 | 889 | Reference |  |  |  |
| M1 | 20 | 4.254 (2.468-7.334) | < 0.001 |  |  |
| **Pathological N stage** | 1050 |  | < 0.001 |  |  |
| N1 | 355 | Reference |  |  |  |
| N0 | 504 | 0.511 (0.347-0.753) | < 0.001 | 0.991 (0.531-1.851) | 0.977 |
| N2 | 116 | 1.288 (0.777-2.135) | 0.327 | 1.365 (0.518-3.597) | 0.530 |
| N3 | 75 | 2.141 (1.211-3.788) | 0.009 | 2.992 (1.175-7.614) | **0.022** |
| **Pathological T stage** | 1066 |  | < 0.001 |  |  |
| T2 | 622 | Reference |  |  |  |
| T1 | 276 | 0.750 (0.499-1.125) | 0.164 | 1.554 (0.749-3.224) | 0.237 |
| T3 | 135 | 1.179 (0.751-1.850) | 0.475 | 0.940 (0.466-1.897) | 0.864 |
| T4 | 33 | 2.815 (1.552-5.105) | < 0.001 | 0.849 (0.319-2.264) | 0.744 |
| **Menopause** | 993 |  | 0.005 |  |  |
| Pre | 228 | Reference |  |  |  |
| Post | 691 | 2.181 (1.313-3.624) | 0.003 | 0.929 (0.429-2.013) | 0.853 |
| Indeterminate | 34 | 1.408 (0.668-2.968) | 0.369 | 0.647 (0.231-1.809) | 0.406 |
| Peri | 40 | 0.384 (0.051-2.879) | 0.352 | 0.521 (0.068-4.021) | 0.532 |
| **PAM50** | 1069 |  | 0.019 |  |  |
| LumA | 558 | Reference |  |  |  |
| Her2 | 79 | 2.264 (1.327-3.864) | 0.003 | 0.603 (0.231-1.574) | 0.301 |
| LumB | 200 | 1.670 (1.093-2.553) | 0.018 | 0.737 (0.406-1.335) | 0.313 |
| Normal | 39 | 1.511 (0.693-3.298) | 0.299 | 0.913 (0.259-3.217) | 0.887 |
| Basal | 193 | 1.281 (0.831-1.976) | 0.262 | 0.771 (0.327-1.821) | 0.553 |
| **Risk score** | 1069 | 5.112 (3.345-7.812) | < 0.001 | 3.636 (1.970-6.711) | **< 0.001** |

**Supplementary Table S3.** Functional annotations of GO/KEGG terms.

| **Biological process (BP)** | | **Cellular component (CC)** | |
| --- | --- | --- | --- |
| GO:0070268 | Cornification | GO:0062023 | Collagen-containing extracellular matrix |
| GO:0019730 | Antimicrobial humoral response | GO:0001533 | Cornified envelope |
| GO:0007586 | Digestion | GO:0043025 | Neuronal cell body |
| GO:0007631 | Feeding behavior | GO:0097060 | Synaptic membrane |
| GO:0023061 | Signal release | GO:0034702 | Ion channel complex |
| GO:0022600 | Digestive system process | GO:0045095 | Keratin filament |
| GO:0015837 | Amine transport | GO:0016324 | Apical plasma membrane |
| GO:0010466 | Negative regulation of peptidase activity | GO:0031225 | Anchored component of membrane |
| GO:0009914 | Hormone transport | GO:1902495 | Transmembrane transporter complex |
| GO:0006865 | Amino acid transport | GO:0005882 | Intermediate filament |
| GO:0050886 | Endocrine process | **Molecular function (MF)** | |
| GO:1990868 | Response to chemokine | GO:0004252 | Serine-type endopeptidase activity |
| GO:1990869 | Cellular response to chemokine | GO:0048018 | Receptor ligand activity |
| GO:0070098 | Chemokine-mediated signaling pathway | GO:0015267 | Channel activity |
| **KEGG term** | | GO:0022803 | Passive transmembrane transporter activity |
| hsa04080 | Neuroactive ligand-receptor interaction | GO:0005179 | Hormone activity |
| hsa03320 | PPAR signaling pathway | GO:0061135 | Endopeptidase regulator activity |
| hsa05033 | Nicotine addiction | GO:0030594 | Neurotransmitter receptor activity |
| hsa04950 | Maturity onset diabetes of the young | GO:0008509 | Anion transmembrane transporter activity |
| hsa04974 | Protein digestion and absorption | GO:0005216 | Ion channel activity |
|  |  | GO:0061134 | Peptidase regulator activity |
